# Effect of bar jump height on kinetics and kinematics of take-off in agility dogs

**DOI:** 10.1371/journal.pone.0315907

**Published:** 2025-01-24

**Authors:** Leena Inkilä, Anna Boström, Pedro Valadão, Janne Avela, Simon Walker, Johanna Mäkitaipale, Anna Bergh, Heli K. Hyytiäinen

**Affiliations:** 1 Faculty of Veterinary Medicine, Department of Equine and Small Animal Medicine, University of Helsinki, Helsinki, Finland; 2 Faculty of Sport and Health Sciences, NeuroMuscular Research Center, University of Jyväskylä, Jyväskylä, Finland; 3 Department of Clinical Sciences, Swedish University of Agricultural Sciences, Uppsala, Sweden; University College Dublin, IRELAND

## Abstract

Sport-related injuries have been reported to occur in around one-third of agility dogs. Higher bar height in competitions has been shown to increase odds of an injury. This study evaluated the effect of bar height on the kinetics and kinematics at take-off to a bar jump. Forces from fore- and hindlimb pairs were measured with force plates. A three-dimensional motion capture system was used to measure sagittal joint kinematics of the shoulder, elbow, carpus, hip, stifle, and tarsal joints, as well as limb coordination, trunk horizontal velocity, take-off distance, and take-off angle. Data were collected for 17 Border Collies at three different bar heights: 80%, 100%, and 120% of wither height. A linear mixed model was used for statistical analysis. At higher bar height, decelerative impulses were greater and accelerative impulses decreased along with greater vertical impulses from forelimb and hindlimb pairs (p<0.001). Post-hoc analyses revealed differences between all three bar heights (p<0.01), except for forelimb decelerative impulse, which did not differ between 80% and 100% heights. Sagittal range of motion was greater, through increased peak flexion or extension, at 120% bar height than at lower bar heights (p<0.05) in almost all measured limb joints. The only exceptions were leading forelimb shoulder and elbow joints and leading hindlimb hip joint. With increasing bar height, the horizontal velocity of trunk decreased (p<0.001), and take-off angle became steeper (p<0.001), with all bar heights differing from each other (p<0.01). Temporal synchronicity between trailing and leading limbs increased and craniocaudal distance decreased in forelimbs (p<0.05) and hindlimbs (p<0.01) as bar height increased. Increased vertical and decelerative impulses, as well as the greater peak flexion and extension angles of joints, may indicate greater load on the tissues at higher bar heights, which could explain the increased odds of injury at higher bar heights in agility dogs.

## Introduction

Approximately one-third of agility dogs suffer a sport-related injury during their sport career [1–3], with the bar jump, A-frame, and dog walk being the obstacles most often associated with injuries [1,2,4,5]. Moreover, bar jumps are the most common obstacle on agility courses [1]. Thus, the highly frequent exposure and the reported association with injuries make bar jumps an important factor to consider in relation to agility dogs’ safety and welfare.

In agility, dogs are divided into four height categories based on their wither height according to the Fédération Cynologique Internationale (FCI) regulations: small (wither height < 35 cm), medium (35 cm to <43 cm), intermediate (43 cm to <48 cm), and large (≥48 cm) [6]. In Europe, larger dogs jump typically higher bar heights in relation to their wither height (up to 125% of wither height) than most smaller dogs (as low as 72% of wither height) [6,7]. In one study, the odds ratio for musculoskeletal injury was increased in agility dogs who jump obstacles at least five centimeters above their wither height relative to dogs jumping proportionally lower heights [8], although another study did not observe this association [2].

Bar height is a highly debated subject in the sport of agility. In 2018, the maximum bar height of the jump obstacles in all dog height categories was reduced by five centimeters according to the renewed international FCI regulations [9]. For example, the maximum bar height of the “large” category was reduced from 65 cm to 60 cm. In addition, a new, fourth dog height category, “intermediate”, was introduced to FCI regulations in 2023 to reduce bar heights for some dogs who used to compete in the “large” category [6]. Similar rule changes of reducing bar heights and adding height categories have been made to national agility regulations in Europe as well as in agility organizations in the United States [10–12]. Nevertheless, even after the regulation changes, the dogs in the lower end of wither height range in all international height categories still jump at least five centimeters above their wither height [6]. Currently, the decisions for the above-mentioned regulation changes have been based on the sparse scientific evidence as well as anecdotal evidence.

Research on the effect of bar height on joint kinematics at take-off is limited. With increasing bar height, it is known that extension of the tarsus and flexion of the shoulder and elbow joints increase as dogs’ hindlimbs are about to leave the ground [13]. However, previous studies have evaluated joint angles only from still images at one or two time points of take-off [13,14]. Thus, the full range of motion (ROM) and maximum flexion or extension angles during take-off are unknown. Greater vertical velocity is required at take-off to increase the height of the jump [15]. Additionally, higher bar height is associated with decreased horizontal velocity during the flight phase in agility dogs [14]. To our knowledge, neither the approach velocity nor the possible deceleration during the stance phases of take-off during different jump heights have been evaluated.

The roles of fore- and hindlimbs appear to be different at take-off. Roughly 55% of the vertical impulse at take-off is produced by the forelimbs when bar height is 90% of the dogs’ wither height [16]. Forelimbs produce mainly decelerative impulse during take-off, whereas the net impulse from hindlimbs is accelerative [16]. Forelimbs are spatially and temporally further away from each other, whereas hindlimbs take-off more synchronously [16]. No studies exist on how bar height affects kinetics or limb synchronicity at take-off.

The overall aim of this study was to examine the effect of bar height on jumping biomechanics at take-off in intermediate and large agility dogs. The focus was the effect of bar jump height on ground reaction forces (GRFs) and sagittal plane kinematics during the stance phase at take-off. Additionally, limb coordination, horizontal velocity, take-off distance, and take-off angle in relation to bar height were evaluated to provide a more complete understanding of jump performance. We hypothesized that an increase in bar height would lead to increased hip and stifle flexion during take-off, and therefore, greater ROM in both joints. We expected the vertical and decelerative impulses exerted on hindlimbs to increase with bar height. Additionally, the take-off angle was expected to be steeper and horizontal velocity at lift-off slower as bar height increases.

## Materials and methods

The study design was approved by the Viikki Campus Research Ethics Committee (statement 10/2019). Dog owners were given written and oral information about the study, and they provided signed consent for participation of their dog.

### Animals

Border Collies competing in agility at different levels were included in the study. Participants were recruited through social media and local agility clubs. Dogs were excluded if they had had an injury or illness causing a break from agility in the past two months prior to data collection. Prior to the jump-related measurements, a veterinary orthopedic surgeon (JM) conducted physical and orthopedic examinations on all dogs. Dogs were excluded if they were not considered fit to perform (e.g. heart murmur, lameness, painful reactions during manipulation of joints).

Power analysis, based on mean values and standard deviations of joint angles at two phases of take-off at two bar heights (93% and 151% of wither height) reported by Birch & Lesniak (2013) (13), showed that 25 dogs would be a sufficient sample size to reveal significant differences (alpha: 0.05, power: 0.8) in joint angles of the shoulder, elbow, stifle, and tarsus between bar heights.

### Experimental setup

**Fig 1:**
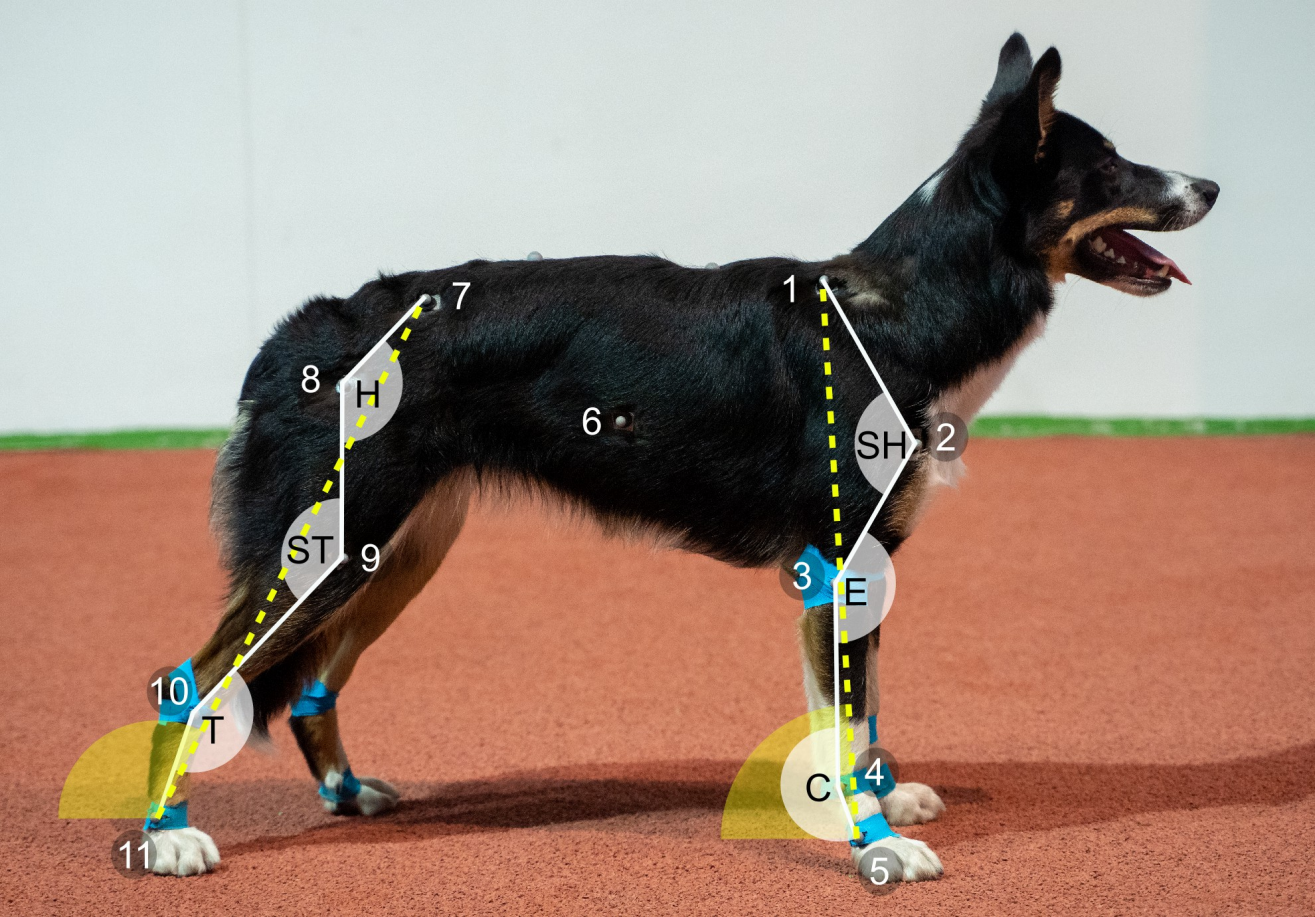
Placement of reflective markers and calculated sagittal joint angles. The markers shown in the image were used to calculate the marked joint angles during stance phases of each limb in take-off. 1 = dorsal border of scapula, 2 = greater tubercle of humerus, 3 = lateral humeral epicondyle, 4 = ulnar styloid process, 5 = distal end of fifth metacarpal, 6 = over the 11^th^ rib at the level of the shoulder joint, 7 = cranial dorsal iliac spine, 8 = greater trochanter of femur, 9 = lateral femoral epicondyle, 10 = lateral malleolus of fibula, 11 = distal end of fifth metatarsal. SH = shoulder, E = elbow, C = carpus, H = hip, ST = stifle, T = tarsus. Dashed yellow line marks the line used for limb angle calculation.

After the physical and orthopedic examinations, measurement of body mass (kg) and wither height (cm) took place. Reflective markers (diameter 9.5 mm) were attached to anatomical landmarks presented in Fig 1 (adapted from [17]). The skin was shaved at the marker sites and markers attached with double-sided tape. The markers on the limbs were additionally fixed with kinesiology tape (Sensiplast, Delta-Sport Handelskontor, Hamburg, Germany).

Dogs performed bar jumps at three bar heights: at 80%, 100%, and 120% of their wither height. These bar heights correspond to the variability in proportional bar heights in current international competition rules. The order of the bar heights was block randomized: there were six possible orders, each of which occurred once in the block of six dogs [18].

Each trial consisted of three consecutive bar jumps of the same height on a straight line with six-meter distances between jumps to achieve course-like speed and striding. Since the order effect may cause kinematic and kinetic differences in the sequence of three jumps, only the take-off of the second jump was measured, representing a course situation with an obstacle before and after each jump. The start line was set five meters before the first jump. A toy or food reward was placed five meters after last jump to ensure the dog’s direction of movement, encouraging it to remain on a straight path throughout the performance. The handler left the dog to wait in the start point and moved past the last jump before releasing the dog with a verbal cue. The handler was instructed to maintain consistent handling throughout all trials. Prior to data collection, handlers were informed about the jump sequence and their dogs’ bar heights and instructed to familiarize their dog with those before the measurements.

A marker-based 3D motion capture system (Vicon Motion Systems, Oxford, UK) consisting of 15 cameras (Vero v2.2) with a sampling frequency of 200 Hz was used. Five force plates (models BP60001200-4K and BP9000900-4K, AMTI Inc, Watertown, MA, USA) placed sequentially without gaps (total area covered 0.6 m * 5.7 m) simultaneously recorded the horizontal and vertical ground reaction forces during take-off at 1 KHz. The surface, including the force plates, was a non-slippery tartan track (3M, St. Paul, MN, USA).

For a valid trial, the dog had to clear all three jumps without visually apparent deviation from the straight line. The aim was to record three valid trials at each bar height with kinematic data recorded of all markers and force plate data of all four limbs. Kinetics were measured only if all paws took off from the force plates. After each trial, contact with the force plate was confirmed from force-plate data and slow-motion video recorded by a mobile phone (OnePlus 5T, OnePlus Technology, Shenzhen, China) at 60 Hz. All valid trials were included in the analysis even if data was missing for some variables (e.g. kinematic data from all markers, but no kinetic data due to dog not properly contacting force plates). Thus, more than three trials per height were included from some dogs. The unbalanced number of trials per height per dog was accounted for in statistical analysis.

### Processing of kinematic and gait variables

A 4^th^-order low-pass Butterworth filter was used on raw marker trajectories (20 Hz) and on the force plate data (150 Hz). Cut-off frequencies were based on evaluation of periodograms. The Cartesian coordinate system had the y-axis positive in the direction of travel, z-axis positive upwards, and x-axis positive towards the dog’s right.

We measured the stance phase of each limb before the aerial phase leading the dog over the obstacle. Touch-down (TD) and lift-off (LO) timings (beginning and end of the stance phase) were recorded for each limb. Force plate data alone were insufficient to determine TD and LO timings if multiple paws contacted the force plate simultaneously. Thus, a combination of force plate data, coordinates of markers on the 5^th^ metacarpal and metatarsal bones, and 3D visualization was used. All recordings were processed by the same person (LI) with excellent intra-rater reliability: intraclass correlation coefficient (two-way mixed effects absolute agreement) was 0.988 (95% confidence interval 0.95–1.00) for forelimb events and 0.998 (95% confidence interval 0.99–1.00) for hindlimb events. During take-off, in gallop, the dogs’ limbs were defined according to their order of contact with the ground as follows: trailing forelimb (TrFL), leading forelimb (LeFL), trailing hindlimb (TrHL), and leading hindlimb (LeHL). If, at take-off, the hindlimbs of the dogs touched the ground exactly at the same time, the hindlimb on the side of the leading forelimb was marked as the trailing hindlimb to allow for comparison with other trials. This approach was chosen as the dogs used a rotary gallop stride pattern in most of the trials, and thus, the leading forelimb and trailing hindlimb were typically ipsilateral.

Sagittal joint angles (i.e. intersegmental angle) were calculated from marker trajectory data using a custom-made 2D kinematic model with Vicon ProCalc software (Vicon Motion Systems, Oxford, UK). The sagittal plane was defined as the plane created by y- (craniocaudal) and z-axes (vertical). Sagittal ROM, maximum flexion, and extension angles were calculated for shoulder, elbow, carpal, hip, stifle, and tarsal joints of trailing and leading fore- and hindlimbs (Fig 1). Joint angles were evaluated throughout the stance phase. To track the movement of the trunk, the kinematic model included a virtual marker ‘Trunk’, which was created midway between the two reflective markers on the sides of the trunk. Additional kinematic variables are listed in Table 1. Each kinematic variable was calculated only if the marker data required for that variable were available. If the marker had fallen off or was poorly visible to the cameras, variables calculated using that marker were not recorded.

**Table 1:** Description of kinematic variables measured during take-off to a jump.

Variable	Description
Joint peak flexion ^a^	Minimum sagittal joint angle during stance.
Joint peak extension ^a^	Maximum sagittal joint angle during stance.
Joint ROM ^a^	Range of motion during stance.
Take-off distance	Distance between jump obstacle and LeHL at TD.
Trunk angle at lift-off	Angle of dog’s trunk, measured from between shoulders to between hips, at LO of LeHL. Relative to horizontal plane.
Take-off angle	Virtual trunk marker’s direction of travel during first 50 ms of the aerial phase, relative to horizontal plane.
Trunk height at TrFL TD	Height of virtual trunk marker, relative to ground, at touch-down of trailing forelimb. Normalized to dog’s wither height.
Trunk height at apex	Highest point of virtual trunk marker, relative to ground, during aerial phase. Value was calculated from vertical velocity immediately after LeHL left the ground. Normalized to dog’s wither height.
Bar clearance	Bar height subtracted from trunk height at the apex. Normalized to dog’s wither height.
Horizontal velocity at approach	Horizontal velocity of virtual trunk marker before TD of TrFL.
Horizontal velocity after LO	Horizontal velocity of virtual trunk marker after LO of LeHL.
Stance time ^b^	Duration of ground contact.
Synchronicity of FLs/HLs	Calculated as time between TD of trailing and leading limbs. Value recorded as percentage of trailing limb stance time [16].
Craniocaudal distance between FLs/HLs	Distance between trailing and leading limbs at TD in y-direction.
Craniocaudal distance between LeFL and TrHL	Distance between LeFL and TrHL at TD in y-direction.
Mediolateral distance between FLs/HLs	Distance between trailing and leading limbs at TD in x-direction.
Craniocaudal distance between HL and trunk marker	Distance between hindlimb paw and virtual trunk marker at TD in y-direction. Positive values indicate paw being caudal to the trunk marker.
Limb angle at TD ^b^	Angle between the ground and the line from dorsal scapula to distal metacarpal bone in FLs or the line from greater trochanter to distal metatarsal bone in HLs in sagittal plane measured at TD of each limb (Fig 1).
Limb angle at LO ^b^	As ‘Limb angle at TD’ but measured at LO.
Stride number	Number of strides before take-off (between jumps 1 and 2).

ROM = range of motion, TrFL = trailing forelimb, LeFL = leading forelimb, TrHL = trailing hindlimb, LeHL = leading hindlimb, TD = touch-down of a limb, LO = lift-off of a limb.

^a^ Joint angles were calculated for shoulder, elbow, carpus, hip, stifle, and tarsal joints with separate values for trailing and leading limbs.

^b^ Separate values for TrFL, LeFL, TrHL, and LeHL.

### Processing of kinetic variables

**Fig 2:**
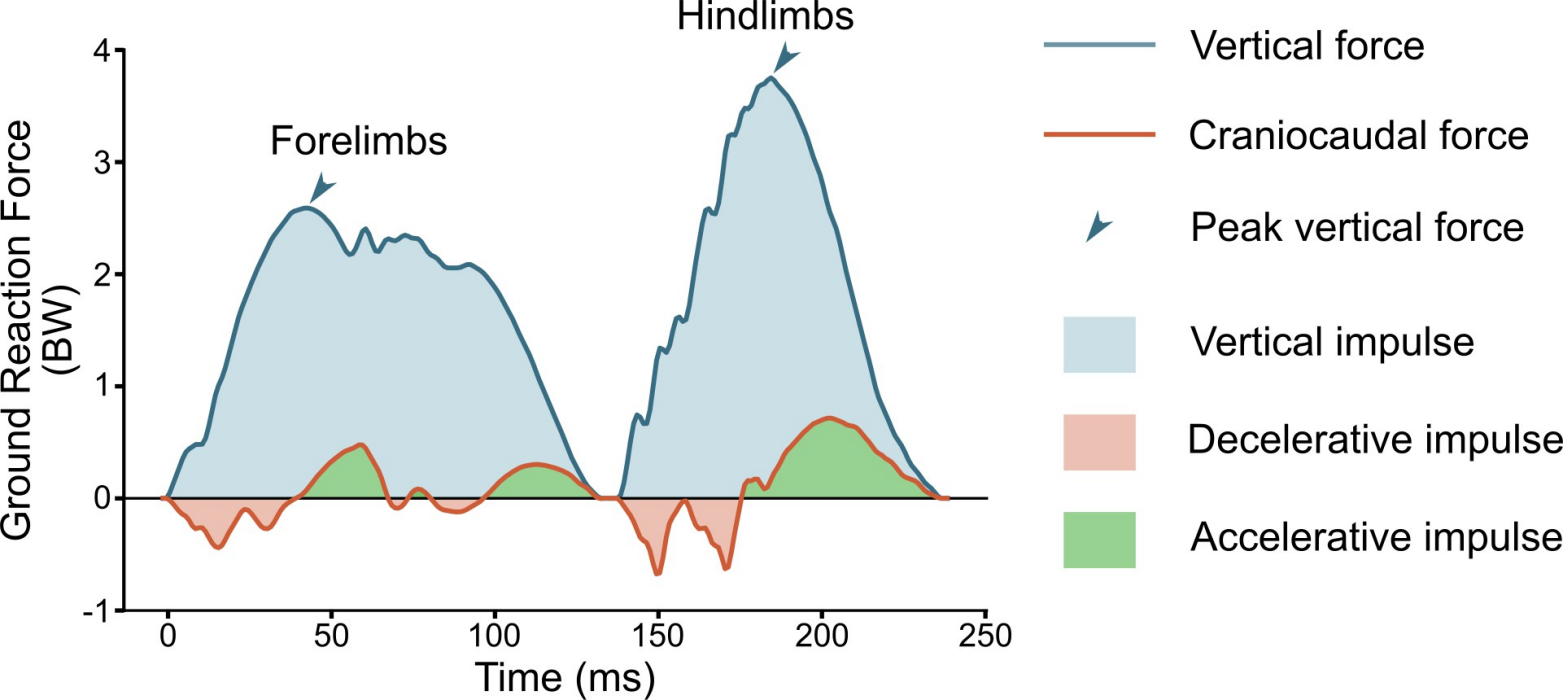
Calculation of peak force and impulse values from force plate data. Peak force and impulse values were calculated separately for fore- and hindlimbs. If trailing and leading limbs contacted separate force plates, force values from the two plates were summed. In this example of individual trial at 100% of wither height, the force curves from fore- and hindlimbs do not overlap, as there had been a suspension phase between fore- and hindlimbs. An example with overlap is presented in S1 Fig.

Vertical and craniocaudal mean forces, peak forces, and impulses were calculated from the force plate data (Fig 2). Force-related values were normalized to body weight (BW). Thus, the unit of force becomes BW and of impulse BWs. The combined force data was used from forelimb pair and hindlimb pair, as the size and placement of the force plates did not allow measurement of individual limb forces. Additionally, vertical and net cranio-caudal impulses were calculated for all four limbs (fore- and hindlimbs combined). Weight distribution was calculated as the proportion of total vertical impulse produced by forelimbs. The direction of the resultant force vector relative to horizontal was calculated from mean vertical and mean craniocaudal forces.

If the leading forelimb and trailing hindlimb simultaneously contacted the same force plate, their force curves overlapped. The magnitude of overlap was assessed by the value of vertical force at the cut-off point. To estimate fore- and hindlimb impulses from these trials, the lowest vertical force value was used as a cut-off; force values before the cut-off timing were used to calculate forelimb impulses and values after the cut-off timing to calculate hindlimb impulses (S1 Fig). To evaluate whether this produced error in calculated impulse values, artificial overlapping versions were created from trials with no overlap and fore- and hindlimb kinetic data available. Twenty artificial versions with increasing overlap were created from each trial (range of overlap magnitude 0.002–2.031 BW). From the artificial overlap versions, the error in the impulse values was calculated by comparing true impulse values from the actual data with no overlap to predicted impulse values from the artificial versions with varying degree of overlap.

The preliminary effect of bar height on impulse values was evaluated from trials without overlap using the same statistical methods as in the final analyses (see Statistical analyses). The smallest expected difference in impulse values between two bar heights with p<0.1 was used for evaluations for each impulse variable. This p-value was chosen to ensure that we evaluated effect of overlap on all variables that might be affected by bar height. Absolute errors below 30% of the expected effect were considered acceptable. The artificial data with overlap magnitude below 0.4 BW (632 artificial trials) had a median overlap of 0.23 BW (interquartile range 0.14–0.32 BW). From all artificial trials with overlap magnitude below 0.4 BW, absolute errors in impulse values were always below 30% of the expected bar height effect for all impulse variables. Mean absolute errors ranged from 0.1% to 5.4% of the expected bar height effect depending on the impulse variable. Thus, we consider the chosen threshold as a good trade-off between the number of acceptable trials and error generated by our method to deal with overlap.

Impulses, mean force values, and impulse distributions for fore- and hindlimbs were discarded from trials where overlap was above 0.4 BW, and only combined impulses for all four limbs were recorded from these trials. Peak force values from these trials were not discarded, as they were not affected by the overlap issue. The same cut-off method was used if the leading hindlimb of the approach stride was simultaneously on the same force plate with the trailing forelimb of the take-off stride. If overlap magnitude was above 0.4 BW, the forelimb impulses and mean force values were discarded along with impulse distributions and combined impulse values for all four limbs.

### Statistical analysis

Descriptive statistics are presented as mean ± standard deviation (SD). The association between ‘Stride number’ and ‘Bar height’ was analyzed with Chi-squared test. A linear mixed model with an unstructured correlation structure, identity link function, and normal distribution was used to evaluate the effect of ‘Bar height’ on previously described kinetic and kinematic variables. Preliminary evaluation showed associations between ‘Stride number’ and dependent variables, leading to its inclusion in the model. Experience of the dog, evaluated as combination of training years and competition class, has been shown to affect jumping biomechanics in agility dogs [16]. However, adding competition class to the model did not improve it for most of the dependent variables based on the Akaike Information Criterion. Thus, competition class was not included in the final model. The model had bar height and stride number as fixed effects, and the dog ID as a random effect. Significance was set at p<0.05.

Analyses were conducted in R version 4.2.2 (R Core Team) using lmer function from lmerTest package (Kuznetsova et al., 2017). The model was fitted using restricted maximum likelihood estimation and Kenward-Roger method for degrees of freedom. Normality of the residuals was confirmed with evaluation of QQ-plots. Lack of multicollinearity between ‘Bar height’ and ‘Stride number’ was confirmed with variance inflation factor (VIF). Post-hoc pairwise comparisons for ‘Bar height’ and estimated marginal means with standard deviations were run using “emmeans” package. Intraclass coefficient (ICC, i.e. subject variance divided by subject plus residual variance) was calculated for each dependent variable to verify the percentage of the total variance that was explained by the dog ID.

## Results

Twenty-six Border Collies were recruited from whom eight dropped out prior to data collection, mainly due to owner-reported injuries of dogs. Of the 18 dogs participating to data collection, one was excluded during orthopedic examination, leaving a final sample of 17 dogs.

Of the 17 dogs, eight were females and nine males. Mean ± standard deviation for age was 4.2 ± 2.1 years and for wither height 51.8 ± 3.2 cm. Nine dogs competed in the lowest class 1, four in class 2, and four in the highest class 3.

A total of 176 trials were included in the analysis: 56 trials with jump bar at 80%, 63 trials at 100% and 57 trials at 120% of wither height. The mean number of analyzed trials per bar height per dog was 3.5 ± 1.4 (range 0–7). Except for two dogs, all dogs had minimum of three analyzed trials on all three bar heights. One dog had only one trial at one bar height, while another dog had minimum of three trials at two bar heights. Twelve trials from eight dogs were excluded because the dog knocked off a bar in the trial sequence; of these trials, 11 (92%) were at 120% bar height and one (8%) at 80% bar height.

The simultaneous contact of the leading forelimb and trailing hindlimb on a force plate resulted in overlapping force curves in 55 trials. In eight of these trials, the overlap magnitude was ≥ 0.4 BW, leading to exclusion of fore- and hindlimb impulses, mean force values, and impulse distributions. Additionally, in 18 trials the leading hindlimb of approach stride overlapped with the trailing forelimb of take-off stride. From five trials with overlap magnitude ≥ 0.4 BW, the following variables were excluded: forelimb impulses and mean force values as well as impulse distributions and combined impulse values from all four limbs.

Full results of the linear mixed model for the fixed effects of bar height and stride number as well as the pairwise comparisons are presented in Tables 2–4 and S1–S6.

**Table 2:** Linear mixed model results: Effect of bar height and approach stride number on kinetics at take-off to a jump in agility dogs.

Variable			Main effects				Estimated marginal mean ± SE			ICC
			Bar height		Stride number		Bar height:			
		n	p-value	Difference	p-value	Difference	80%	100%	120%	%
**Forelimbs**										
	Mean vertical force (BW)	158	<0.001	80<100<120	<0.001	2<1	1.71 ± 0.03	1.79 ± 0.03	1.89 ± 0.03	73
	Mean craniocaudal force (BW)	158	<0.001	120<100<80	<0.001	2<1	0.01 ± 0.02	-0.02 ± 0.02	-0.12 ± 0.02	62
	Peak vertical force (BW)	167	<0.001	80<100<120	0.980		2.79 ± 0.09	3.02 ± 0.09	3.35 ± 0.09	69
	Vertical impulse (BWs)	158	<0.001	80<100<120	<0.001	2<1	0.207 ± 0.005	0.216 ± 0.005	0.235 ± 0.005	66
	Decelerative impulse (BWs)^a^	158	<0.001	120<80, 120<100	0.170		-0.012 ± 0.002	-0.014 ± 0.002	-0.022 ± 0.002	49
	Accelerative impulse (BWs)	158	<0.001	120<100<80	<0.001	2<1	0.014 ± 0.001	0.011 ± 0.001	0.007 ± 0.001	50
	Net craniocaudal impulse (BWs)	158	<0.001	120<100<80	<0.001	2<1	0.001 ± 0.002	-0.003 ± 0.002	-0.015 ± 0.002	58
	Direction of resultant force vector (°)	158	<0.001	120<100<80	<0.001	2<1	90.4 ± 0.5	89.2 ± 0.5	86.4 ± 0.5	61
**Hindlimbs**										
	Mean vertical force (BW)	163	<0.001	80<100<120	0.002	2<1	1.96 ± 0.05	2.06 ± 0.05	2.26 ± 0.05	73
	Mean craniocaudal force (BW)	163	<0.001	120<100<80	<0.001	2<1	0.19 ± 0.02	0.14 ± 0.02	0.08 ± 0.02	75
	Peak vertical force (BW)	171	<0.001	80<100<120	<0.001	2<1	3.53 ± 0.07	3.66 ± 0.07	3.82 ± 0.07	76
	Vertical impulse (BWs)	163	<0.001	80<100<120	<0.001	2<1	0.182 ± 0.003	0.188 ± 0.003	0.207 ± 0.003	43
	Decelerative impulse (BWs)^a^	163	<0.001	120<100<80	0.718		-0.009 ± 0.001	-0.011 ± 0.001	-0.014 ± 0.001	69
	Accelerative impulse (BWs)	163	<0.001	120<100<80	<0.001	2<1	0.026 ± 0.001	0.023 ± 0.001	0.021 ± 0.001	63
	Net craniocaudal impulse (BWs)	163	<0.001	120<100<80	<0.001	2<1	0.017 ± 0.002	0.012 ± 0.002	0.007 ± 0.002	68
	Direction of resultant force vector (°)	163	<0.001	120<100<80	<0.001	2<1	95.4 ± 0.5	93.7 ± 0.5	92.0 ± 0.5	65
**All four limbs**										
	Vertical impulse (BWs)	163	<0.001	80<100<120	<0.001	2<1	0.391 ± 0.007	0.405 ± 0.007	0.443 ± 0.007	59
	Net craniocaudal impulse (BWs)	163	<0.001	120<100<80	<0.001	2<1	0.018 ± 0.003	0.008 ± 0.003	-0.008 ± 0.003	54
	Weight distribution (% of vertical impulse on FLs)	156	0.340		0.011	2<1	53.0 ± 0.6	53.5 ± 0.6	53.2 ± 0.6	60

SE = standard error, ICC = intraclass correlation coefficient, BW = body weight, FLs = forelimbs.

Main effects for bar height and stride number are presented as p-values along with estimated marginal means for all bar heights. Different shade of orange background indicates significant difference (p<0.05) between bar heights. Darker background denotes greater force, acceleration, or deceleration. White background indicates no differences between bar heights. All p-values of pairwise comparison for bar height are presented in S1 Table. Estimated marginal means for stride numbers are presented in S2 Table.

^a^ Lower values indicate greater deceleration.

**Table 3:** Linear mixed model results: Effect of bar height and approach stride number on jump arch and limb coordination at take-off in agility dogs.

Variable			Main effects				Estimated marginal mean ± SE			ICC
			Bar height		Stride number		Bar height:			
		n	p-value	Difference	p-value	Difference	80%	100%	120%	%
**Horizontal velocity at approach** (m/s)		161	<0.001	120<100<80	<0.001	2<1	7.27 ± 0.11	7.15 ± 0.11	6.86 ± 0.11	81
**Horizontal velocity after lift-off** (m/s)		173	<0.001	120<100<80	<0.001	2<1	7.29 ± 0.14	7.11 ± 0.14	6.60 ± 0.14	80
**Take-off distance** (cm)		174	0.012	120<80, 100<80	<0.001	2<1	192 ± 9	183 ± 9	183 ± 9	81
**Trunk angle at lift-off** (°)		161	<0.001	80<100<120	0.001	2<1	15.0 ± 0.7	16.8 ± 0.7	20.9 ± 0.7	59
**Take-off angle** (°)		154	<0.001	80<100<120	0.071		10.7 ± 0.6	12.9 ± 0.6	17.2 ± 0.5	61
**Trunk height at TrFL touch-down** (% of wither height)		162	<0.001	120<100<80	0.017	1<2	76.2 ± 0.7	75.1 ± 0.6	73.6 ± 0.6	69
**Trunk height at the apex** (% of wither height)		173	<0.001	80<100<120	<0.001	2<1	128.9 ± 3.2	141.8 ± 3.2	168.3 ± 3.2	71
**Bar clearance** (% of wither height)		173	<0.001	100<120, 100<80	<0.001	2<1	48.9 ± 3.2	41.8 ± 3.2	48.3 ± 3.2	71
**Stance time**										
	Trailing forelimb (ms)	176	<0.001	80<120, 100<120	0.799		82 ± 3	84 ± 3	91 ± 3	83
	Leading forelimb (ms)	176	<0.001	80<120, 100<120	<0.001	2<1	75 ± 2	77 ± 2	80 ± 2	71
	Trailing hindlimb (ms)	176	<0.001	80<120, 100<120	0.525		79 ± 2	80 ± 2	84 ± 2	73
	Leading hindlimb (ms)	176	<0.001	80<120, 100<120	0.778		74 ± 2	75 ± 2	80 ± 2	64
**Synchronicity**										
	Forelimbs (% of TrFL stance time)^a^	176	<0.001	120<100<80	0.010	2<1	56.4 ± 1.9	54.2 ± 1.9	50.3 ± 1.9	58
	Hindlimbs (% of TrHL stance time)^a^	176	<0.001	120<100<80	<0.001	2<1	22.7 ± 2.4	19.7 ± 2.4	13.1 ± 2.4	73
**Distance between limbs at TD**										
	Craniocaudal distance between FLs (cm)	173	<0.001	120<100<80	<0.001	2<1	35.2 ± 1.3	33.9 ± 1.3	31.8 ± 1.3	67
	Craniocaudal distance between HLs (cm)	173	<0.001	120<100<80	<0.001	2<1	13.5 ± 1.5	11.5 ± 1.4	7.3 ± 1.5	74
	Craniocaudal distance between LeFL and TrHL (cm)	173	<0.001	120<100<80	<0.001	2<1	12.6 ± 1.3	11.1 ± 1.2	7.4 ± 1.2	59
	Mediolateral distance between FLs (cm)	173	<0.001	80<120, 100<120	0.462		8.0 ± 0.7	8.4 ± 0.6	9.4 ± 0.7	73
	Mediolateral distance between HLs (cm)	173	<0.001	80<120, 100<120	<0.001	1<2	12.7 ± 0.5	13.0 ± 0.5	14.2 ± 0.5	76
	Craniocaudal distance between TrHL and trunk marker (cm)	173	<0.001	120<80, 120<100	<0.001	1<2	6.3 ± 0.6	5.9 ± 0.5	4.4 ± 0.5	57
	Craniocaudal distance between LeHL and trunk marker (cm)	174	<0.001	120<80, 120<100	<0.001	1<2	5.9 ± 0.5	5.4 ± 0.5	4.1 ± 0.5	41
**Limb angle**										
	TrFL at touch-down (°)	170	<0.001	120<100<80	0.005	1<2	72.8 ± 0.9	71.3 ± 0.9	68.5 ± 0.9	67
	TrFL at lift-off (°)	173	<0.001	120<100<80	<0.001	2<1	131.0 ± 0.8	129.9 ± 0.8	128.0 ± 0.8	68
	LeFL at touch-down (°)	173	<0.001	120<100<80	<0.001	1<2	71.4 ± 0.6	69.9 ± 0.6	67.5 ± 0.6	42
	LeFL at lift-off (°)	174	<0.001	120<100<80	<0.001	2<1	118.6 ± 1.0	116.5 ± 1.0	112.5 ± 1.0	67
	TrHL at touch-down (°)	173	<0.001	120<80, 120<100	0.030	1<2	66.6 ± 0.6	65.7 ± 0.5	62.9 ± 0.6	41
	TrHL at lift-off (°)	175	<0.001	120<100<80	<0.001	2<1	127.3 ± 0.7	125.2 ± 0.7	121.8 ± 0.7	62
	LeHL at touch-down (°)	174	<0.001	120<80, 120<100	<0.001	1<2	65.6 ± 0.7	65.0 ± 0.7	63.3 ± 0.7	48
	LeHL at lift-off (°)	176	<0.001	120<100<80	0.018	2<1	123.9 ± 0.9	121.5 ± 0.9	119.1 ± 0.9	69

CI = confidence interval, ICC = intraclass correlation coefficient, TD = touch-down, TrFL = trailing forelimb, LeFL = leading forelimb, TrHL = trailing hindlimb, LeHL = leading hindlimb.

Main effects for bar height and stride number are presented as p-values along with estimated marginal means for all bar heights. Different shade of orange background indicates significant difference (p<0.05) between bar heights. Darker background denotes higher value. White background indicates no differences between bar heights. All p-values of pairwise comparison for bar height are presented in S3 Table. Estimated marginal means for stride numbers are presented in S4 Table.

^a^ Lower values indicate greater synchronicity.

**Table 4:** Linear mixed model results: Effect of bar height and approach stride number on sagittal joint kinematics at take-off to a jump in agility dogs.

Variable			Main effects				Estimated marginal mean ± SE			ICC
			Bar height		Stride number		Bar height:			
		N	p-value	Difference	p-value	Difference	80%	100%	120%	%
**Trailing forelimb**										
	Shoulder peak flexion (°)	167	0.23		<0.001	1<2	115.0 ± 1.5	114.6 ± 1.5	114.1 ± 1.5	82
	Shoulder peak extension (°)	167	<0.001	80<120, 100<120	<0.001	1<2	128.2 ± 2.1	129.2 ± 2.1	131.2 ± 2.1	88
	Shoulder ROM (°)	167	<0.001	80<120, 100<120	0.67		13.3 ± 1.4	14.7 ± 1.4	17.2 ± 1.4	68
	Elbow peak flexion (°)	168	<0.001	120<100<80	0.003	1<2	124.5 ± 2.0	122.7 ± 2.0	119.2 ± 2.0	78
	Elbow peak extension (°)	168	0.014	120<80	0.134		159.5 ± 1.2	158.4 ± 1.2	157.6 ± 1.2	66
	Elbow ROM (°)	168	<0.001	80<120, 100<120	0.058		34.8 ± 1.0	35.5 ± 1.0	38.1 ± 1.0	48
	Carpus peak flexion (°)	171	0.872		0.746		184.5 ± 2.3	184.2 ± 2.2	184.7 ± 2.2	77
	Carpus peak extension (°)	171	0.002	80<120, 100<120	0.213		234.7 ± 2.9	235.6 ± 2.9	238.1 ± 2.9	84
	Carpus ROM (°)	171	0.007	80<120, 100<120	0.148		50.4 ± 1.5	51.6 ± 1.5	53.7 ± 1.5	48
**Leading forelimb**										
	Shoulder peak flexion (°)	171	0.040	80<100	0.251		118.8 ± 2.0	120.1 ± 1.9	119.7 ± 1.9	89
	Shoulder peak extension (°)	171	0.355		0.183		140.6 ± 2.1	140.9 ± 2.1	141.5 ± 2.1	86
	Shoulder ROM (°)	171	0.151		<0.001	2<1	21.6 ± 0.9	20.7 ± 0.9	21.7 ± 0.9	57
	Elbow peak flexion (°)	171	0.180		0.002	1<2	124.6 ± 2.3	125.8 ± 2.3	124.3 ± 2.3	81
	Elbow peak extension (°)	171	0.343		0.557		162.5 ± 1.3	163.4 ± 1.2	162.9 ± 1.2	68
	Elbow ROM (°)	171	0.460		<0.001	2<1	37.7 ± 1.6	37.5 ± 1.5	38.4 ± 1.5	70
	Carpus peak flexion (°)	172	0.413		0.218		187.3 ± 1.9	187.6 ± 1.9	186.5 ± 1.9	74
	Carpus peak extension (°)	172	0.017	80<100, 80<120	<0.001	2<1	230.2 ± 3.1	233.0 ± 3.1	232.5 ± 3.1	84
	Carpus ROM (°)	172	0.018	80<100, 80<120	<0.001	2<1	43.1 ± 2.1	45.7 ± 2.1	46.3 ± 2.1	63
**Trailing hindlimb**										
	Hip peak flexion (°)	172	<0.001	120<80, 120<100	<0.001	2<1	129.8 ± 1.9	129.7 ± 1.9	127.4 ± 1.9	80
	Hip peak extension (°)	172	0.625		0.003	2<1	171.5 ± 1.5	171.9 ± 1.5	172.2 ± 1.5	71
	Hip ROM (°)	172	<0.001	80<120, 100<120	0.141		41.6 ± 1.1	42.1 ± 1.1	44.7 ± 1.1	63
	Stifle peak flexion (°)	172	0.647		0.722		130.4 ± 1.4	130.7 ± 1.4	129.8 ± 1.4	50
	Stifle peak extension (°)	172	<0.001	80<120, 100<120	0.949		155.9 ± 1.3	156.1 ± 1.3	157.6 ± 1.3	84
	Stifle ROM (°)	172	0.001	80<120, 100<120	0.107		25.3 ± 0.9	25.2 ± 0.8	26.7 ± 0.8	31
	Tarsus peak flexion (°)	172	<0.001	120<80, 120<100	<0.001	1<2	107.6 ± 1.8	106.0 ± 1.8	102.9 ± 1.8	65
	Tarsus peak extension (°)	172	0.945		0.595		174.6 ± 1.3	174.4 ± 1.3	174.4 ± 1.3	82
	Tarsus ROM (°)	172	<0.001	80<120, 100<120	<0.001	2<1	67.0 ± 1.2	68.4 ± 1.2	71.5 ± 1.2	47
**Leading hindlimb**										
	Hip peak flexion (°)	172	0.172		0.618		131.2 ± 2.4	132.2 ± 2.4	132.6 ± 2.4	85
	Hip peak extension (°)	172	0.022	80<120	0.054		168.2 ± 2.4	169.1 ± 2.4	170.4 ± 2.4	85
	Hip ROM (°)	172	0.534		0.010	1<2	37.1 ± 1.4	36.9 ± 1.4	37.7 ± 1.4	60
	Stifle peak flexion (°)	172	<0.001	120<80, 120<100	0.101		135.0 ± 1.4	135.8 ± 1.4	133.1 ± 1.4	67
	Stifle peak extension (°)	172	0.857		0.021	2<1	161.1 ± 1.3	160.9 ± 1.3	160.9 ± 1.3	84
	Stifle ROM (°)	172	<0.001	80<120, 100<120	0.801		26.0 ± 0.8	25.0 ± 0.8	27.8 ± 0.8	48
	Tarsus peak flexion (°)	172	<0.001	120<80, 120<100	0.437		107.7 ± 1.7	109.2 ± 1.7	105.3 ± 1.7	60
	Tarsus peak extension (°)	172	0.002	80<120, 100<120	0.350		175.2 ± 1.0	176.0 ± 1.0	176.9 ± 1.0	71
	Tarsus ROM (°)	172	<0.001	80<120, 100<120	0.178		67.4 ± 1.6	66.7 ± 1.6	71.5 ± 1.6	60

N = number of trials, CI = confidence interval, ICC = intraclass correlation coefficient, ROM = range of motion.

Main effects for bar height and stride number are presented as p-values along with estimated marginal means for all bar heights. Different shade of orange background indicates significant difference (p<0.05) between bar heights. Darker background denotes greater flexion, extension, or ROM. White background indicates no differences between bar heights. All p-values of pairwise comparisons for bar height are presented in S5 Table. Estimated marginal means for stride numbers are presented in S6 Table.

### Effect of bar height on peak and mean forces at take-off

Bar height affected peak and mean forces exerted on fore- and hindlimbs (Table 2). In fore- and hindlimbs, there was a main effect of bar height for mean and peak vertical forces (p<0.001). Furthermore, the post-hoc analysis revealed that all bar heights differed from each other (p<0.001 for all pairwise comparisons). In forelimbs, the estimated differences between 80% and 120% heights were a 0.19 BW (body weight) increase in mean vertical force and a 0.56 BW increase in peak vertical force. In hindlimbs, the estimated differences between 80% and 120% heights were a 0.30 BW increase in mean vertical force and a 0.30 BW increase in peak vertical force.

Bar height affected mean craniocaudal force of the fore- and hindlimbs (p<0.001), with significant differences between all bar heights in both limb pairs (p<0.001). The estimated difference in mean craniocaudal force between 80% and 120% heights was -0.13 BW in forelimbs and -0.10 BW in hindlimbs. In both fore- and hindlimbs, the direction of the resultant force vector was affected by bar height (p<0.001), with all heights differing from each other (p<0.001 for all pairs). The estimated difference between 80% and 120% heights was -4.0° in forelimbs and -3.4° in hindlimbs, indicating a more backward-oriented direction of the force vector at 120% height.

### Effect of bar height on impulses at take-off

Impulse values in fore- and hindlimbs were affected by bar height (Table 2). There was a main effect of bar height on vertical impulse of fore- and hindlimbs (p<0.001), with differences emerging between all bar heights (80% vs. 100%, p = <0.001 in FLs and p = 0.008 in HLs, p<0.001 for other pairwise comparisons). The vertical impulse increased by an estimated 0.028 BWs in forelimbs and by 0.024 BWs in hindlimbs from 80% to 120% height.

**Fig 3:**
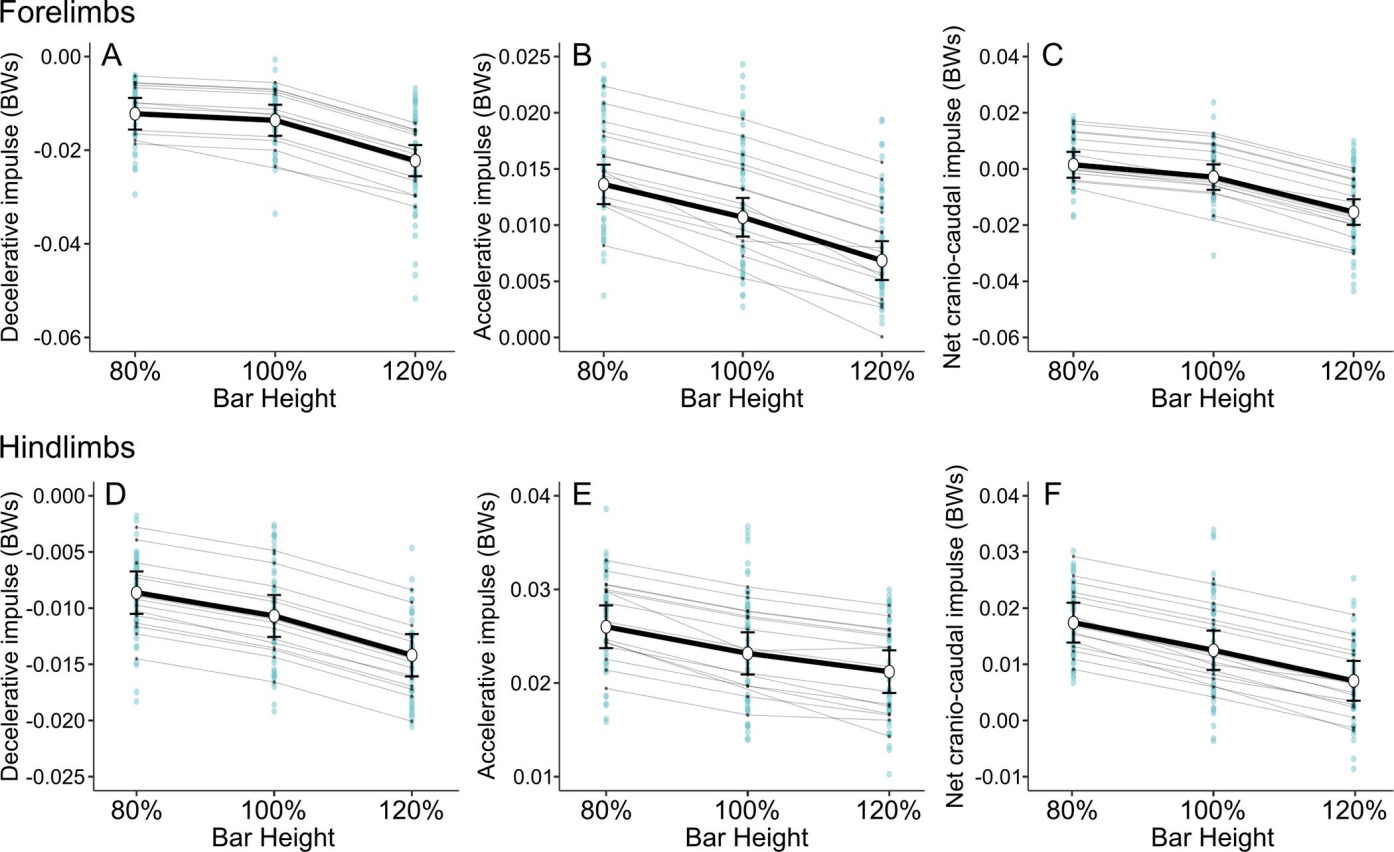
Craniocaudal impulses of fore- and hindlimbs at three bar heights during take-off. Estimated marginal means are presented as white dots along with 95% confidence interval lines. Light blue dots represent the observed values and light gray lines the random effects. A-C: Decelerative, accelerative, and net craniocaudal impulses produced by forelimbs. D-F: Decelerative, accelerative, and net craniocaudal impulses produced by hindlimbs. In both fore- and hindlimbs, all bar heights differed significantly from each other in all craniocaudal impulses (p≤0.001), except for decelerative impulse (A), which did not significantly differ between 80% and 100% bar heights in forelimbs.

Bar height affected the decelerative impulse of forelimbs (p<0.001), with post-hoc analyses showing greater deceleration as height increased (Fig 3); differences were between 80% and 120% heights (β = -0.010 BWs, p<0.001) and 100% and 120% heights (-0.009 BWs, p<0.001). The decelerative impulse of hindlimbs was affected by bar height (p<0.001), with all bar heights differing from each other in post-hoc analyses (p<0.001) (Fig 3). The estimated difference in decelerative impulse between 80% and 120% heights was -0.006 BWs. Bar height had an effect on accelerative impulse in fore- and hindlimbs (p<0.001), with all heights differing from each other (p**≤**0.001 for all pairwise comparisons) (Fig 3). The estimated decrease in accelerative impulse between 80% and 120% heights was -0.007 BWs in forelimbs and -0.005 BWs in hindlimbs.

The net craniocaudal impulse of fore- and hindlimbs was affected by bar height (p<0.001), with post-hoc analyses showing differences between all bar heights (p**≤**0.001 for all pairwise comparisons) (Fig 3). The estimated difference between 80% and 120% heights was -0.017 BWs in forelimbs and -0.010 BWs in hindlimbs. The net craniocaudal impulse decreased from accelerative to decelerative in forelimbs and remained accelerative in hindlimbs at all bar heights.

There was a main effect of bar height on total vertical impulse produced during take-off by all four limbs (p<0.001). All bar heights differed from each other in post-hoc analyses (p**≤**0.001). The estimated difference between 80% and 120% heights was 0.052 BWs. The net craniocaudal impulse was affected by bar height (p<0.001), with all bar heights differing from each other (p<0.001). The estimated difference between 80% and 120% heights in net craniocaudal impulse was -0.026 BWs, shifting the net impulse from accelerative to decelerative.

### Effect of bar height on jump arch and velocity

Bar height affected the jump arch and velocity of the dog (Table 3). The height of the trunk at touch-down of trailing forelimb was affected by the bar height (p<0.001), with post-hoc analyses showing differences between all bar heights (p<0.001 for all pairwise comparisons). The estimated difference between 80% and 120% heights was -2.6% of wither height. There was a main effect of bar height for take-off distance (p = 0.012), with differences between 80% and 100% heights (β = -8 cm, p = 0.010) and 80% and 120% heights (β = -9 cm, p = 0.009).

There was a main effect of bar height on trunk height at the apex of jump (p<0.001), with all bar heights differing from each other (p<0.001). The estimated increase between 80% and 120% heights was 39.4% of wither height. However, there was also a main effect of bar height on bar clearance (p<0.001), with post-hoc analyses showing differences between 80% and 100% heights (β = -7.2%, p<0.001) and 100% and 120% heights (β = 6.6%, p<0.001). Thus, at 100% height the bar clearance was less than at other heights, with no difference between 80% and 120% heights.

Horizontal velocity at approach, just before touch-down of trailing forelimb, was affected by bar height (p<0.001) with differences between all bar heights (p = 0.003 for 80% vs. 100% comparison, p<0.001 for other comparisons). Additionally, there was a main effect of bar height on horizontal velocity after lift-off (p<0.001), with all three bar heights differing from other heights (p<0.001 for all pairwise comparisons). The estimated difference in horizontal velocity between 80% and 120% heights was -0.41 m/s at approach and -0.69 m/s after lift-off.

### Effect of bar height on limb coordination

Positioning and timing of limbs were affected by bar height (Table 3). Stance times were longer in all limbs at 120% height. In trailing and leading forelimbs, bar height had an effect on stance time (p<0.001 for both forelimbs), with differences between 80% and 120% heights (p<0.001 for both forelimbs) and 100% and 120% heights (p<0.001 for TrFL, p = 0.002 for LeFL). The estimated increase in stance time between 80% and 120% heights was 9 ms in TrFL and 5 ms in LeFL. Similarly, in both hindlimbs, there was a main effect of bar height for stance time (p<0.001), with differences between 80% and 120% heights (p<0.001 for both TrHL and LeHL) and 100% and 120% heights (p<0.001 for both TrHL and LeHL). The estimated increase in stance time between 80% and 120% heights was 5 ms in TrHL and 6 ms in LeHL.

**Fig 4:**
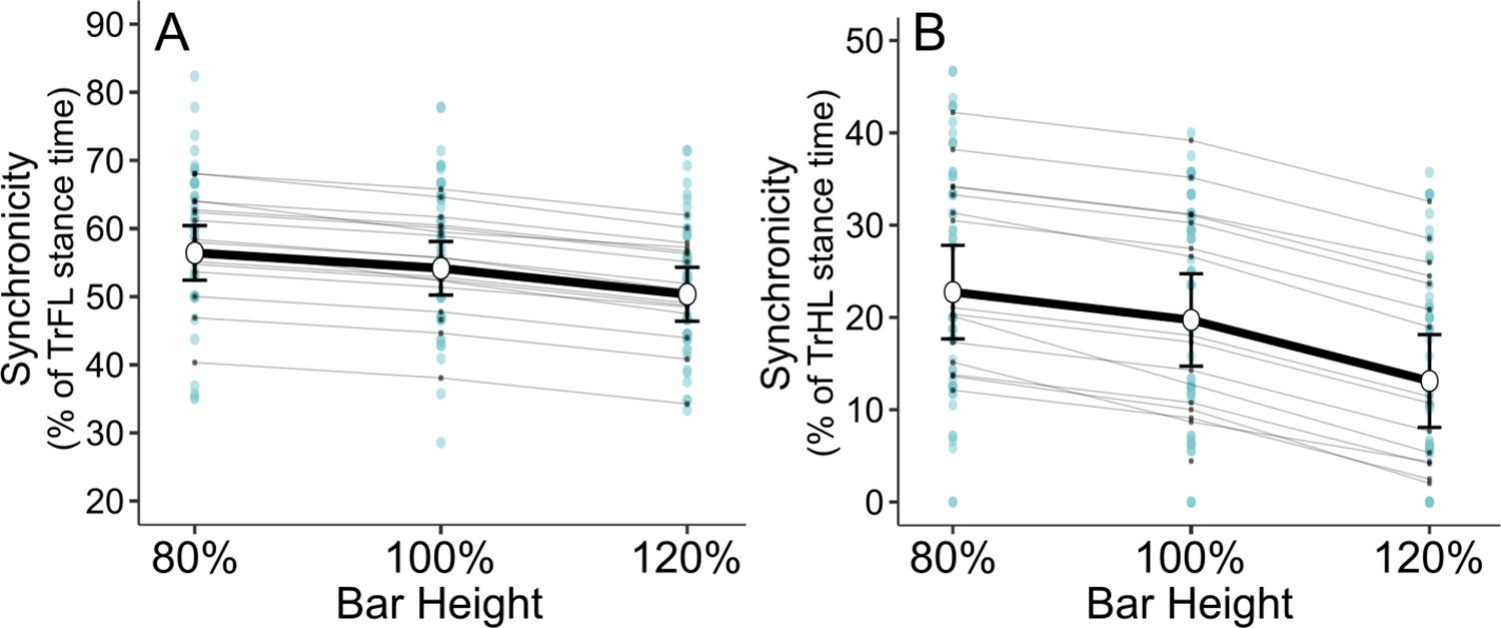
Synchronicity in touch-down timings of trailing and leading forelimbs (A) and hindlimbs (B). Estimated marginal mean is presented as a white dot along with the 95% confidence interval line. Light blue dots represent the observed values and light gray lines the random effects. Synchronicity is presented as the percentage of trailing limb stance time at which leading limb touch-down occurs. Lower value indicates more synchronous touch-down of trailing and leading limbs. In both fore- and hindlimbs, values differed significantly between all bar heights (80% vs. 100%, p = 0.048 in forelimbs and p = 0.006 in hindlimbs, p<0.001 for other pairwise comparisons). TrFL = trailing forelimb, TrHL = trailing hindlimb.

In fore- and hindlimbs, bar height affected limb synchronicity (p<0.001 for both limb pairs) (Fig 4), with differences between all bar heights (80% vs. 100%, p = 0.048 in FLs and p = 0.006 in HLs, p<0.001 for other pairwise comparisons). The estimated difference between 80% and 120% heights was -6.1% of TrFL stance time in forelimbs and -9.6% of TrHL stance time in hindlimbs, with lower values at 120% height indicating greater synchronicity.

The positioning of the paws relative to each other was affected by bar height. There was a main effect of bar height for craniocaudal distance between trailing and leading fore- and hindlimbs (p<0.001). Post-hoc analysis revealed differences between all bar heights (80% vs. 120%, p<0.001 in FLs and HLs, 80% vs. 100%, p = 0.037 in FLs and p = 0.003 in HLs, 100% vs. 120%, p**≤**0.001 in FLs and HLs). The estimated difference in paw distance between 80% and 120% heights was -3.5 cm in forelimbs and -6.2 cm in hindlimbs. The craniocaudal distance between trailing forelimb and leading hindlimb was additionally affected by bar height (p<0.001), with differences emerging between all bar heights (80% vs. 100%, p = 0.039, p<0.001 for other pairwise comparisons). The estimated difference between 80% and 120% heights was -5.1 cm, indicating that fore- and hindlimbs were closer to each other at 120% height.

Mediolateral distance between trailing and leading limbs was affected by bar height in fore- and hindlimbs (p<0.001 for both limb pairs), with differences between 80% and 120% heights (p<0.001 for both limb pairs) and 100% and 120% heights (p<0.001 for both limb pairs). The estimated increase in mediolateral paw distance between 80% and 120% heights was 1.4 cm in forelimbs and 1.5 cm in hindlimbs.

### Effect of bar height on limb angle

In all four limbs, the limb angle was always below 90° (vertical) at touch-down and above 90° at lift-off. In all limbs, the limb angle decreased at both touch-down and lift-off as bar height increased (Table 3). The limb angle of the trailing forelimb was affected by the bar height both at touch-down and at lift-off (p<0.001), with all three heights differing from each other (p = 0.002 at TD and p = 0.003 at LO 80% vs. 100%, p<0.001 for other pairwise comparisons at TD and LO). The estimated difference between 80% and 120% heights was -4.3° at touch-down and -3.1° at lift-off. Similarly, there was a main effect of bar height on limb angle of leading forelimb at touch-down (p<0.001) and lift-off (p<0.001), with differences between all bar heights (p≤0.001 at both TD and LO). The estimated difference between 80% and 120% heights was -3.9° at touch-down and -6.2° at lift-off.

In the trailing hindlimb, the bar height affected limb angle at touch-down (p<0.001) and lift-off (p<0.001). At touch-down, post-hoc analyses revealed differences between 80% and 120% heights (β = -3.6°, p<0.001) and 100% and 120% heights (β = -2.8°, p<0.001). At lift-off, all three bar heights differed from each other (p<0.001), with the estimated difference between 80% and 120% heights being -5.5°. Additionally, the bar height affected limb angle of leading hindlimb at touch-down (p<0.001) and lift-off (p<0.001). At touch-down, differences were observed between 80% and 120% heights (β = -2.3°, p<0.001) and 100% and 120% heights (β = -1.7, p = 0.001). At lift-off, all three bar heights differed from each other (p<0.001), with an estimated difference between 80% and 120% heights of -4.9°.

### Effect of bar height on sagittal joint kinematics in forelimbs

**Fig 5:**
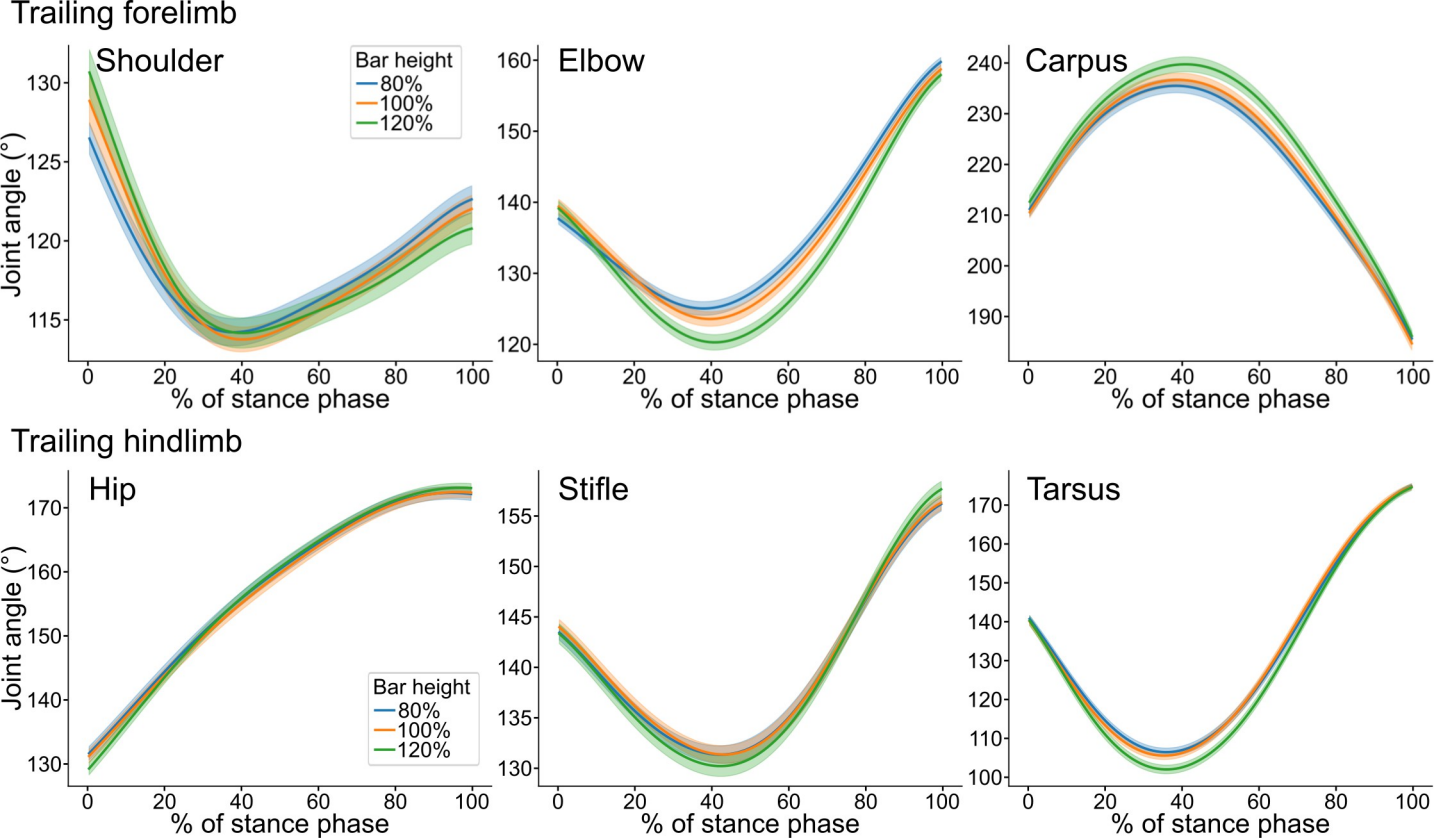
Sagittal joint angles during stance phase at take-off to a jump. Joint angles of trailing forelimb shoulder, elbow, and carpus and trailing hindlimb hip, stifle, and tarsus are presented. Mean curves ± standard error of mean from all trials at three bar heights are shown: 80% (blue), 100% (orange), and 120% (green) of wither height. Figures for leading fore- and hindlimb joints are presented in S2 Fig. Please note that the effect of stride number is not controlled in this figure, and the number of trials per dog per bar height varied from 0 to 7.

The sagittal joint angles of shoulder, elbow, and carpal joints during stance phase at take-off are presented in Figs 5 and S2. Bar height had a significant main effect on peak extension, peak flexion, or ROM of multiple forelimb joints, especially in the trailing forelimb. Full results are presented in Table 4. Most differences were observed between 120% and lower heights, with no significant difference between 80% and 100% heights.

In the trailing forelimb shoulder, there was a main effect of bar height on peak extension and ROM (p<0.001), with post-hoc analyses showing differences between 80% and 120% heights (peak extension β = 3.0°, p<0.001; ROM β = 3.8°, p<0.001) and 100% and 120% heights (peak extension β = 2.0°, p = 0.001; ROM β = 2.5°, p<0.001). In the trailing forelimb elbow, the peak flexion was affected by bar height (p<0.001), with all bar heights differing from each other (p = 0.034 for 80% vs. 100%, p<0.001 for other pairwise comparisons). The estimated difference between 80% and 120% heights was -5.3°.

Additionally, bar height had an effect on peak extension of the trailing forelimb elbow (p = 0.014), but post-hoc analyses showed a difference only between 80% and 120% heights (β = -1.9°, p = 0.004). Trailing forelimb elbow ROM was affected by bar height (p<0.001), with differences between 80% and 120% heights (β = 3.4, p<0.001) and 100% and 120% heights (β = 2.7, p<0.001).

In the carpal joint of the trailing forelimb, bar height had a main effect on peak extension (p = 0.002) and ROM (p = 0.007). Post-hoc analyses revealed differences between 80% and 120% heights (peak extension β = 3.4°, p<0.001; ROM β = 3.3°, p = 0.002) and 100% and 120% heights (peak extension β = 2.5°, p = 0.008; ROM β = 2.1°, p = 0.039).

In the leading forelimb, peak extension and carpal joint ROM were affected by bar height (p = 0.017 and p = 0.018, respectively), with differences between 80% and 100% heights (peak extension β = 2.8°, p = 0.007; ROM β = 2.6°, p = 0.025) and 80% and 120% heights (peak extension β = 2.3°, p = 0.027; ROM β = 3.1°, p = 0.008).

### Effect of bar height on sagittal joint kinematics in hindlimbs

Sagittal joint angles of hip, stifle, and tarsal joints during stance phase at take-off are presented in Figs 5 and S2. There was an effect of bar height on peak extension, peak flexion, and ROM of multiple joints in both hindlimbs. The full results are presented in Table 4. In both hindlimbs, joint kinematics differed at the 120% height compared with the lower heights, whereas no differences were observed between 80% and 100% heights.

In the trailing hindlimb, peak flexion and ROM of hip joint were affected by bar height (p<0.001), with differences between 80% and 120% heights (peak flexion β = -2.4°, p<0.001; ROM β = 3.2°, p<0.001) and 100% and 120% heights (peak flexion β = -2.3°, p = 0.001; ROM β = 2.6°, p<0.001).

There was main effect of bar height on peak extension and ROM of the trailing hindlimb stifle (p<0.001). Post-hoc comparisons revealed significant differences between 80% and 120% heights (peak extension β = 1.7°, p<0.001; ROM β = 2.4°, p = 0.002) and 100% and 120% heights (peak extension β = 1.6°, p<0.001; ROM β = 2.4°, p = 0.001).

In the tarsus of the trailing hindlimb, bar height had an effect on peak flexion (p<0.001) and ROM (p<0.001), with post-hoc analyses showing differences between 80% and 120% heights (peak flexion β = -4.6°, p<0.001; ROM β = 4.5°, p<0.001) and 100% and 120% heights (peak flexion β = -3.1°, p = 0.001; ROM β = 3.1°, p<0.001).

In the hip joint of leading hindlimb, the bar height had an effect on peak extension (p = 0.022), with the only difference being between 80% and 120% heights (β = 2.2°, p = 0.006). Stifle peak flexion and ROM were also affected by bar height (p<0.001), with differences between 80% and 120% heights (peak flexion β = -2.0°, p = 0.009; ROM β = 1.8°, p = 0.002) and 100% and 120% heights (peak flexion β = -2.8°, p<0.001; ROM β = 2.8°, p<0.001).

In the tarsal joint of the leading hindlimb, there was a main effect of bar height on peak flexion, peak extension, and ROM (p≤0.002). For all variables, the pairwise differences were observed between 80% and 120% heights (peak flexion β = -2.4°, p = 0.016; peak extension β = 1.7°, p<0.001; ROM β = 4.1°, p<0.001) and 100% and 120% heights (peak flexion β = -3.9°, p<0.001; peak extension β = 0.9°, p = 0.045; ROM β = 4.8°, p<0.001).

### Effect of approach stride number

In 118 in 176 trials (67%), dogs took one stride between obstacles 1 and 2 (one-stride approach), with the remaining trials utilizing a two-stride approach. No association between ‘Bar height’ and ‘Stride number’ (p = 0.74) was observed. There was individual variability in striding: 9 of the 17 dogs always used a one-stride approach, three dogs always a two-stride approach, and the remaining five dogs varied their striding.

When using a one-stride approach, dogs lowered the trunk before take-off (β = -1.5% of wither height, p = 0.017) and took off further away from the obstacle (β = 74 cm, p<0.001) with greater bar clearance (β = 17.3% of wither height, p<0.001) compared to a two-stride approach. Horizontal velocity after lift-off was greater with one-stride approach (β = 0.36 m/s, p<0.001). Fore- and hindlimbs were less synchronous (FLs: β = 4.7% of TrFL stance time, p = 0.010; HLs: β = 6.9% of TrHL stance time, p<0.001) when using a one-stride approach.

When using a one-stride approach, vertical impulses were greater in forelimbs (β = 0.044 BWs, p<0.001) and hindlimbs (β = 0.027 BWs, p<0.001) than with the two-stride approach. The weight was shifted more towards forelimbs with a one-stride approach (β = 1.4%, p = 0.011). The net craniocaudal impulse of all four limbs was greater with a one-stride approach (β = 0.016 BWs, p<0.001), indicating greater horizontal acceleration. Full results regarding the effect of approach stride number on the biomechanics at take-off are presented in Tables 3, 4, S2, S4 and S6.

## Discussion

Increasing bar height resulted in multiple biomechanical adaptations at take-off to jump in agility dogs. The vertical and decelerative impulses produced by fore- and hindlimb pairs increased and accelerative impulses decreased with higher bar height. Bar height did not affect weight distribution between fore- and hindlimbs. The horizontal velocity of the dog decreased and take-off angle became steeper with increasing bar height, as hypothesized. As bar height increased, the temporal synchronicity increased in fore- and hindlimbs with decreased craniocaudal distances between the paws. In most measured limb joints, the ROM was greater at 120% than at lower bar heights through greater peak flexion, peak extension, or both. The expected increase in hip peak flexion during take-off was observed only in trailing hindlimbs, and the expected increase in stifle peak flexion appeared only in the leading hindlimb. However, peak flexion increased in both tarsal joints at 120% bar height.

### Kinetics

The effect of bar height on kinetics at take-off in agility dogs has not, to our knowledge, been reported previously. Increased total vertical impulse was required to jump over higher bars. Longer stance phases in all limbs allowed vertical forces to be produced over prolonged duration of time as bar height increased. The dogs in this study may have used this strategy to limit increase in peak vertical forces. Whether peak vertical forces of individual limbs were increased, could not be evaluated in this study.

Fore- and hindlimbs contributed equally to the increase in total vertical impulse produced during take-off with no change in weight distribution between fore- and hindlimbs. Around 53% of the total vertical impulse was produced by forelimbs at all bar heights, which is similar to previous reports of 55–56% in take-off to agility jump (bar height 90% of wither height) and 56–58% in galloping dogs [16,19–21].

The net craniocaudal impulse produced during take-off shifted from accelerative to decelerative through increased decelerative and decreased accelerative impulses from fore- and hindlimbs. Similar results have been reported in hindlimbs of horses at take-off [22]. This tactic may be used to redirect the mainly horizontal movement at approach into an increasingly vertical direction as bar height increases. In our study, the net craniocaudal impulse produced by hindlimbs remained accelerative at all heights, highlighting the role of hindlimbs in production of accelerative forces, as reported in galloping dogs [21].

The increased vertical and decelerative impulses associated with higher bar heights could increase the load on dogs’ musculoskeletal tissues, predisposing to injuries—although this data does not allow to evaluate how these were altered in individual limbs. Repeated production of greater impulses during agility course and training sessions may lead more rapidly to fatigue when using high bar heights. In humans, acute fatigue reduces postural control and muscle strength, which in turn can increase injury risk [23]. In agility dogs, injuries occur more often towards the end of the training session or competition day, suggesting that fatigue may also contribute to injuries in agility dogs [2].

In humans, decelerations are associated with predominantly eccentric muscles actions [24]. In agility dogs, *musculus biceps brachii* and *musculus supraspinatus* show peak activations at the beginning of stance phase at take-off [25], when decelerative forces are produced and when shoulder joint is, according to our data, flexing. Eccentric muscle actions are associated with high muscle forces, which are transferred to the skeleton via tendons. This repetitive tensile loading may contribute to biceps and supraspinatus tendinopathies, which are among the most common injuries in agility dogs [26] and thought to result from overuse [27]. Higher bar heights may increase these loads through greater decelerative and vertical forces. However, the decelerative forces appear to be higher at take-off to a turning jump than in jumping in a straight line [16,28]. Whether decelerative forces of turning jumps are also associated with bar height and determination of the magnitudes of deceleration during other tasks on the agility course require further research.

### Horizontal velocity and movement of the trunk

Bar height has been reported to reduce horizontal velocity over the bar jump [14]. Here, we showed that the horizontal velocity was decreased already when approaching the take-off as well as when lifting off into the jump’s aerial phase. The higher the bar, the more kinetic energy has to be transferred into potential energy at take-off. Dogs’ ability to perform this transfer of energy may be reduced at higher approach speeds, and thus, dogs slow down before take-off to a higher jump.

On the other hand, greater speed at lower bar heights, and therefore, greater kinetic energy, may increase the risk of injury in case of accidents, such as collisions or slips, which have been reported to cause injuries in agility [1,2]. Dog speed over faultless agility runs has, however, not been shown to be associated with risk for agility-related injuries [2], whereas in another dog sport, flyball, faster dogs do have higher risk for injuries [29,30]. However, in agility, bar height is probably not the only factor affecting speed over an agility course; for example, course design (number of turns, length of straight lines), surface or handler position may impact speed as well. Future studies should continue to investigate the effect of speed on injury risk in agility dogs.

Movement and position of the trunk were affected by bar height. When bar height increased, dogs lowered slightly their trunk when coming into take-off and after lift-off continued at a steeper angle. As expected, the calculated trunk height at the apex of the jump increased with bar height. However, the clearance between the trunk and bar was the least at 100% and greater at 80% and 120%. Thus, the height of the actual jump at 80% and 100% heights were quite close to each other, whereas the dogs jumped markedly higher at 120% bar height. This may explain why the two lower heights were often biomechanically less different to each other, and the highest height required the greatest biomechanical adjustments in the jump performance of the dog. As higher jump requires greater vertical force production at take-off to achieve higher vertical velocity and, therefore, requires more energy, the unnecessarily high bar clearance at 120% was not optimal strategy and increased the demands at this bar height even further. Additionally, knocking off a bar was observed almost exclusively at the highest bar height, suggesting that dogs may have struggled to perform at that height.

### Limb coordination

In our study, the dogs changed their limb positions relative to the obstacle and to each other as bar height changed, whereas in ridden horses limb positions at take-off have been reported to remain relatively constant across difference obstacle heights [31]. In our sample, the take-off point was closer to the obstacle when bar height was at or above 100% of wither height. Dogs are known to adjust jump trajectory to minimize mechanical work [32]. Thus, the reduced take-off distance to higher bar height might be more efficient. Whether the length of the jump trajectory was altered, could not be evaluated in our study, but length of the jump trajectory has been reported to increase up to bar heights 100–125% of wither height with decrease in length when bar height is above 125% of wither height [14]. Additionally, landing distance from the first jump in the sequence, if altered by bar height, could have affected the take-off distance to the second jump, which was analyzed in our study.

Craniocaudal distances between all limbs decreased along with greater synchronicity in fore- and hindlimbs. This positioning of the limbs aids in rotating the trunk into more vertical position and in dividing vertical forces more equally between trailing and leading hindlimbs. At all bar heights, temporal synchronicity appeared to be greater than in high-speed rotary gallop on flat [19] with even more pronounced difference to gallop as bar height increased.

The limb angles at touch-down and lift-off were affected by bar height in our sample. As height increased, forelimbs contacted the ground with a greater protraction angle, thus, further in front of the center of gravity. This positioning is likely to have led to the increased deceleration during stance phases in forelimbs. At lift-off, all limbs were at a more vertical limb position (less retraction) when bar height increased, probably to produce a steeper take-off angle. This is likely to have resulted in shorter duration of the accelerative phase and decreased accelerative impulses.

### Forelimb joint kinematics

Sagittal joint kinematics were altered by bar height in the trailing forelimb shoulder, elbow, and carpus as well as in the carpus of the leading forelimb. In an earlier study, no effect of bar height was observed when forelimb joint angles were measured from a single still frame towards the end of the stance phase [13]. Recording sagittal joint kinematics throughout the stance phase allowed us to observe effects that had not been reported previously. Greater adjustments in joint kinematics were observed in the trailing forelimb as bar height increased. The trailing forelimb has been reported to produce approximately 30% of the total vertical impulse at take-off to a jump, which is a higher percentage than for other limbs [16]. We were not able to record forces for individual limbs, but the trailing forelimb might have had a greater role in producing vertical force than the leading forelimb. It could explain why alterations in joint kinematics were observed mainly in the trailing forelimb. [16] In both forelimbs, the carpal joint showed greater peak extension, which occurred shortly before the mid-stance of take-off. The peak carpal extension was less than that reported in dogs entering A-frame [33] but similar to that of dogs landing from a jump or A-frame [34]. During all of these agility activities, the peak carpal extension is greater than in healthy dogs in trot [35–37]. Carpal joint injuries, such as sprains and hyperextension injuries, have been reported in agility dogs [2,4,26]. Further studies are needed to clarify, whether the estimated two-to-three-degree reduction in carpal extension at low bar heights, occurring at the end of joint’s ROM, results in clinically relevant decrease in load on the carpal joint over repetitions.

### Hindlimb joint kinematics

In both hindlimbs, we observed an effect of bar height on joint kinematics, with greatest ROM in almost all measured hindlimb joints at the 120% bar height. Thus, dogs are required to produce force over a greater ROM and at more extreme joint angles as bar height increases.

At 120% bar height, the trailing hindlimb had greater peak hip flexion by two degrees mean difference, occurring at the beginning of the stance. Hip flexion may have been used to produce the greater protraction of trailing hindlimb at limb touch-down when jumping higher bar heights. The hypothesized greater hip flexion was, however, not observed in the leading hindlimb. The leading hindlimb had greater peak hip extension, although only by 2.2 degrees, at the highest height, which occurred towards the end of the stance. The greater hip extension probably allowed the trunk of the dog to take a more vertical position during lift-off. However, the peak extension values of the hip joint appear to be slightly greater in the trailing hindlimb than in the leading hindlimb. In both hindlimbs, the extension angles were greater than those reported for most breeds in low-speed gallop or in passive ROM measurements [38,39].

When the hip is extended, the *musculus iliopsoas*, one of the most injured muscles in agility dogs [3,4,40], is stretched. When the dog performs a vertical jump from stand position, in which the hindlimb action resembles that of jumping over an obstacle, *m*. *iliopsoas* is activated from beginning to end of the stance phase [41]. As previously described, eccentric muscle actions can cause muscle damage, which increases with greater stretch of the muscle [42]. Extension of the hip, with concurrent stretch and possibly activation of *m*. *iliopsoas*, during the jump take-off may contribute to the chronic injuries of *m*. *iliopsoas*, which are thought to result from repetitive microtrauma [43]. However, bar height only affected extension in the hip joint of the leading hindlimb, whereas the more marked extension of the trailing hindlimb hip joint was unaltered by bar height. Thus, reduction of bar height, at least in the ranges evaluated by this study, might not mitigate the risk for iliopsoas injuries in agility dogs.

In the leading hindlimb, there was greater peak extension of the tarsal joint (end of stance) at the highest height, which has been previously reported when comparing take-off at two bar heights (93% and 151%) [13]. As the tarsal peak extension values in our study were beyond those reported for passive extension and extension during gallop [37,38], even the estimated 1.7-degree difference could be clinically relevant considering the repeated nature of jumping in agility. In our study, at 120% height, both tarsal joints had additionally greater peak flexion, which occurred around the first third of the stance. Similarly, increased peak flexion and ROM of both tarsal joints have been observed with increasing horizontal acceleration in greyhounds on flat ground [44]. As higher jump requires greater vertical velocity at lift-off and, therefore, greater vertical acceleration during take-off, agility dogs may utilize similar strategies to increase vertical acceleration during jumping as greyhounds use on flat to increase horizontal acceleration.

### Other factors affecting jumping biomechanics

Previous studies on jump biomechanics in agility dogs have not reported the number of strides between jumps even when multi-obstacle sequences have been used [14,16,45,46]. The multi-obstacle sequences enable course-like speed and performance, thus allowing evaluation of truly sport-specific movement. For these reasons, we used a sequence of three jumps in our set-up. The number of strides between two jump obstacles varied individually across dogs as well as within dogs in a consistent sequence even for the same bar height. However, no association between the number of strides and bar height was observed. Our study showed that stride number was associated with multiple biomechanical differences in kinetics and kinematics. Thus, it was important to control for the effect of stride number in the statistical model to evaluate the effects of bar height more accurately. Additionally, these results highlight the complexity of the sport; i.e. multiple factors affect the loads put on these dogs even in a simple sequence as in straight-line jumps, with the bar height being just one factor. Future biomechanical studies on agility dogs should report and, if needed, control for striding within the obstacle sequence.

Additionally, the high intraclass correlation coefficients indicate a marked individual variation in the biomechanics of jumping; the random factor accounting for the variability among dogs explained a higher proportion of the variation than stride number or bar height. The highest individual variation was observed in joint peak flexion and peak extension angles, which may be affected by conformation of the dog. Horizontal velocity and take-off distance also varied markedly between individuals.

## Limitations

The sample size calculated through power analyses was not achieved, which may result in type 2 errors (false negatives). Yet almost all hypothesized effects of bar height were confirmed. Additionally, the power calculations were a rough estimate, as methods, evaluated joint angles, and sample of dog breeds differed markedly.

Due to the size and orientation of the force plates, forces from individual limbs could not be measured. Therefore, for example, peak forces could not be calculated for each limb individually. Forces are not distributed evenly to trailing and leading limbs [16] and the combined forces do not provide full information of the loads on individual limbs. The greater temporal synchronicity at higher bar heights has probably contributed to the greater vertical peak forces measured from limb pairs in this study. Additionally, decelerative and accelerative impulses were calculated as a sum of trailing and leading limb forces. Therefore, there were time points where accelerative forces produced by one of the limbs may have been cancelled the decelerative forces produced by another limb. Thus, the values of decelerative and accelerative impulses do not depict the total decelerative or total accelerative forces produced by two limbs. However, the net craniocaudal impulse values are not affected. Future studies should evaluate the forces from individual limbs at take-off to jump obstacles of varying height and possibly investigate net joint moments and limb stiffness as well.

Measured ground reaction forces were associated with other measured variables such as velocity, take-off angle and limb synchronicity, which were not included as covariates in the final statistical model because they were associated with bar height. In this study, we did not aim to fit models that best predicted each dependent variable. Rather, we chose to keep the model simple and easy to interpret and discuss, considering the numerous dependent variables in this study and limited sample size. Our focus was on how alteration of bar height, choice made by humans, affects the dog in a sport-specific environment where the dog can choose to make multiple alterations to its performance in response to the altered bar height.

The highest bar height in our study was probably above the bar height that some of the participating dogs were used to jumping. This was likely especially in dogs whose wither height was at the higher end of their height category (jumping usually lower proportional bar heights) and in younger dogs who had not competed before the regulation changes where jump heights were reduced. Although handlers were informed about their dogs’ individual bar heights beforehand and encouraged to familiarize their dog with the bar heights if needed, it is possible that inexperience in jumping at 120% bar height may have influenced the results.

The sequence of jumps allowed for evaluation of bar height effects in sport-specific setting, but as a result, the observed bar height effects might, to certain degree, be specific to this sequence. Each sequence, or even placement of start line, restricts the choices for the dog: for example, in our setup the dogs were forced to take either one or two strides between two obstacles. Restriction of take-off point has been shown to affect the jump trajectory as dogs aim to minimize the mechanical energy cost [32]. Evaluation of bar height effects in other sequence (e.g. different spacing or approach from tunnel) is recommended for future research.

Skin displacement is known to produce error in marker-based kinematic data in dogs, especially in the proximal joints [47–49]. Unfortunately, there is currently no means to account for this error in jumping dogs. Therefore, the reported absolute joint angle values should be interpreted with caution, especially regarding shoulder and hip joint values. While the same measurement system was used at all bar heights, differences in performance, such as change in velocity, may have affected the degree of skin displacement error at different bar heights.

## Conclusion

The increase in bar height resulted in multiple biomechanical adaptations in jumping performance of agility dogs. Dogs decelerated more, accelerated less, and produced greater vertical impulse during take-off with fore- and hindlimbs when bar height increased. With increasing bar height, limbs were positioned closer to each other in craniocaudal direction with greater temporal synchronicity. Additionally, sagittal ROM of most limb joints was greater at 120% than at lower heights. Increased vertical and decelerative impulses as well as greater peak flexion or extension angles of joints may indicate greater load on tissues at higher bar heights in a straight-line jump sequence, which may contribute to sport-related injuries in agility dogs.

## Supporting information

S1 Fig Calculation of peak force and impulse values from trial with overlap. This figure shows an individual trial at 100% bar height. In some trials leading forelimb and trailing hindlimb contacted same force plate simultaneously, leading to their force curves overlapping with each other. To estimate fore- and hindlimb impulses from these trials, the lowest vertical force value was used as cut-off: Force values before the cut-off timing were used to calculate forelimb impulses and values after it for hindlimb impulses. The magnitude of overlap was assessed by the value of vertical force at the cut-off point. This approach was used only for trials where the magnitude of overlap was below 0.4 BW. In the depicted trial, magnitude of overlap was 0.36 BW.
(TIF)

S2 Fig Sagittal joint angles of during stance phase at take-off to a jump. Joint angles of leading forelimb shoulder, elbow and carpus, and leading hindlimb hip, stifle and tarsus. Mean curves ± standard error of mean from all trials at three bar heights are shown: 80% (blue), 100% (orange) and 120% (green) of wither height.
(TIF)

S1 Table Linear mixed model results: Main effect of bar height and pairwise differences in kinetics at take-off to a jump in agility dogs. (DOCX)

S2 Table Linear mixed model results: Main effect of approach stride number on kinetics at take-off to a jump in agility dogs. (DOCX)

S3 Table Linear mixed model results: Main effect of bar height and pairwise differences on jump arch and limb coordination at take-off to a jump in agility dogs. (DOCX)

S4 Table Linear mixed model results: Main effect of approach stride number on jump arch and limb coordination at take-off to a jump in agility dogs. (DOCX)

S5 Table Linear mixed model results: Main effect of bar height and pairwise differences in sagittal joint kinematics at take-off to a jump in agility dogs. (DOCX)

S6 Table Linear mixed model results: Main effect of approach stride number on sagittal joint kinematics at take-off to a jump in agility dogs. (DOCX)
