# Supplementary figures and images for: Effect of bar jump height on kinetics and kinematics of take-off in agility dogs
Source: PLoS One. 2025 Jan 24;20(1):e0315907. doi: 10.1371/journal.pone.0315907 (PMC11761639; doi:10.1371/journal.pone.0315907)

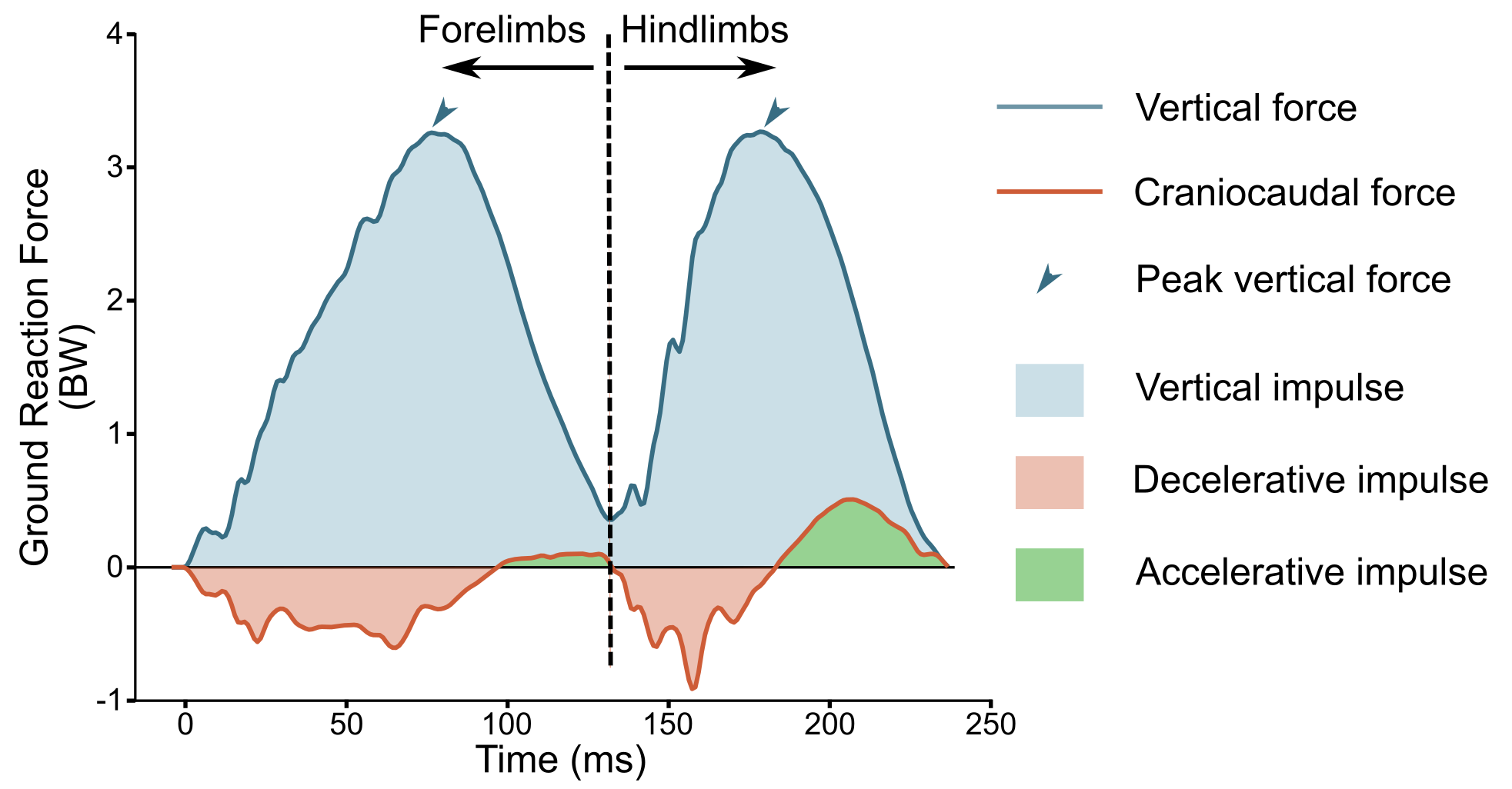

Supplement: S1 Fig — This figure shows an individual trial at 100% bar height. In some trials leading forelimb and trailing hindlimb contacted same force plate simultaneously, leading to their force curves overlapping with each other. To estimate fore- and hindlimb impulses from these trials, the lowest vertical force value was used as cut-off: Force values before the cut-off timing were used to calculate forelimb impulses and values after it for hindlimb impulses. The magnitude of overlap was assessed by the value of vertical force at the cut-off point. This approach was used only for trials where the magnitude of overlap was below 0.4 BW. In the depicted trial, magnitude of overlap was 0.36 BW. (TIF) [file pone.0315907.s001.tif]

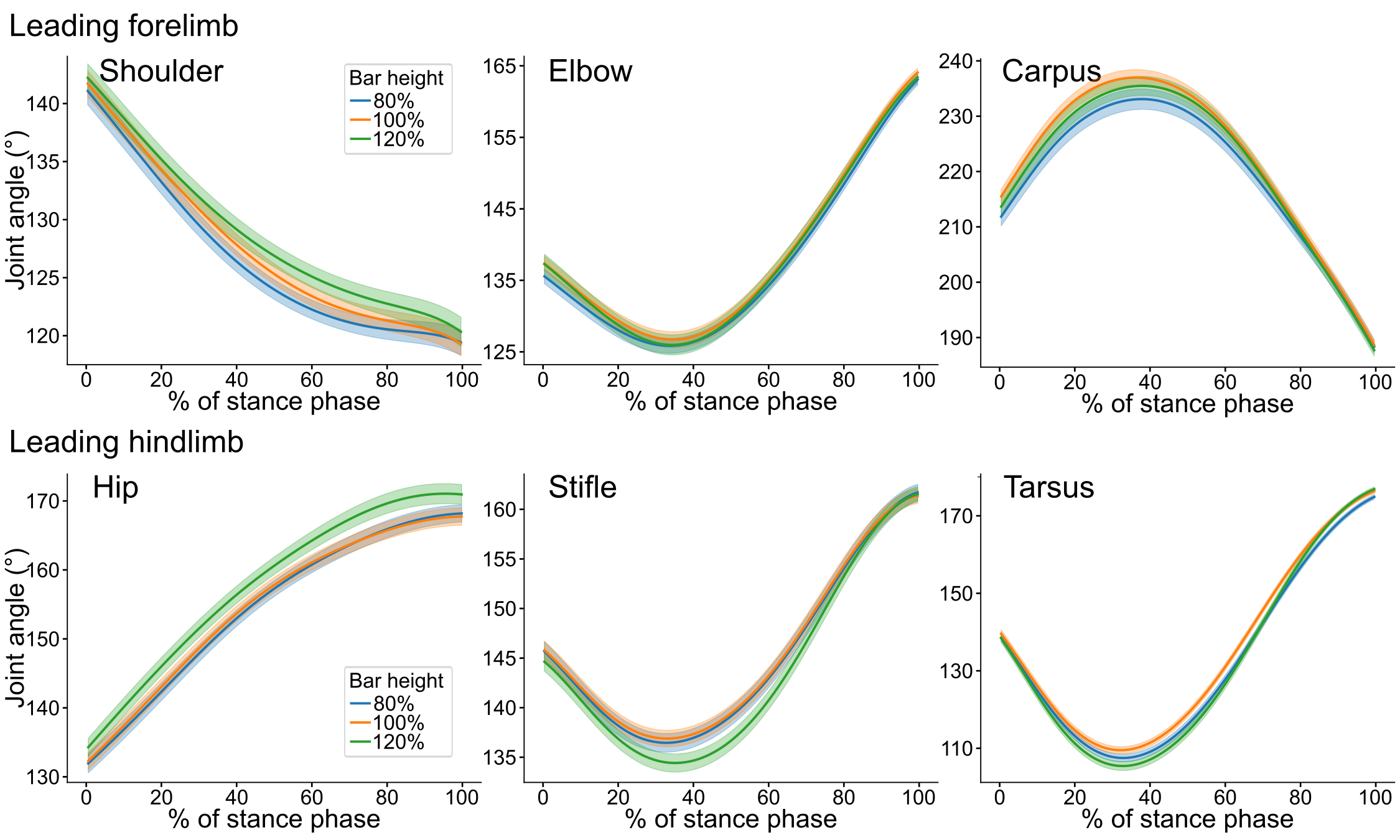

Supplement: S2 Fig — Joint angles of leading forelimb shoulder, elbow and carpus, and leading hindlimb hip, stifle and tarsus. Mean curves ± standard error of mean from all trials at three bar heights are shown: 80% (blue), 100% (orange) and 120% (green) of wither height. (TIF) [file pone.0315907.s002.tif]
