# Supplementary material for: Effect of bar jump height on kinetics and kinematics of take-off in agility dogs
Source: PLoS One. 2025 Jan 24;20(1):e0315907. doi: 10.1371/journal.pone.0315907 (PMC11761639; doi:10.1371/journal.pone.0315907)
Supplement: S1 Table — (DOCX) [file pone.0315907.s003.docx]

**S1 Table. Linear mixed model results: main effect of bar height and pairwise differences in kinetics at take-off to a jump in agility dogs.**

|  | | **Bar height** | **120% - 80%** | | | | **120%-100%** | | | | **100%-80%** | | | |
| --- | --- | --- | --- | --- | --- | --- | --- | --- | --- | --- | --- | --- | --- | --- |
| **Variable** | | **p-value** | **Estimate** | **95% CI** | **SE** | **p-value** | **Estimate** | **95% CI** | **SE** | **p-value** | **Estimate** | **95% CI** | **SE** | **p-value** |
| **Forelimbs** | |  |  |  |  |  |  |  |  |  |  |  |  |  |
|  | Mean vertical force (BW) | <0.001 | 0.19 | 0.15–0.22 | 0.02 | <0.001 | 0.10 | 0.05–0.11 | 0.02 | <0.001 | 0.08 | 0.07–0.14 | 0.02 | <0.001 |
|  | Mean craniocaudal force (BW) | <0.001 | -0.13 | -0.15–(-0.11) | 0.01 | <0.001 | -0.09 | -0.11–(-0.08) | 0.01 | <0.001 | 0.04 | -0.05–(-0.02) | 0.01 | <0.001 |
|  | Peak vertical force (BW) | <0.001 | 0.56 | 0.47–0.65 | 0.04 | <0.001 | 0.33 | 0.25–0.41 | 0.04 | <0.001 | 0.23 | 0.14–0.32 | 0.04 | <0.001 |
|  | Vertical impulse (BWs) | <0.001 | 0.028 | 0.023–0.034 | 0.003 | <0.001 | 0.019 | 0.014–0.024 | 0.003 | <0.001 | 0.009 | 0.004–0.014 | 0.003 | 0.001 |
|  | Decelerative impulse (BWs)^a^ | <0.001 | -0.010 | -0.012–(-0.008) | 0.001 | <0.001 | -0.009 | -0.011–(-0.006) | 0.001 | <0.001 | -0.001 | -0.003–0.001 | 0.001 | 0.213 |
|  | Accelerative impulse (BWs) | <0.001 | -0.007 | -0.008–(-0.005) | 0.001 | <0.001 | -0.004 | -0.005–(-0.003) | 0.001 | <0.001 | -0.003 | -0.004–(-0.002) | 0.001 | <0.001 |
|  | Net craniocaudal impulse (BWs) | <0.001 | -0.017 | -0.020–(-0.014) | 0.001 | <0.001 | -0.012 | -0.015–(-0.010) | 0.001 | <0.001 | -0.004 | -0.007–(-0.002) | 0.001 | 0.001 |
|  | Direction of resultant force vector (°) | <0.001 | -4.0 | -4.6–(-3.4) | 0.3 | <0.001 | -2.8 | -3.4–(-2.2) | 0.3 | <0.001 | -1.2 | -1.8–(-0.6) | 0.3 | <0.001 |
| **Hindlimbs** | |  |  |  |  |  |  |  |  |  |  |  |  |  |
|  | Mean vertical force (BW) | <0.001 | 0.30 | 0.25–0.35 | 0.02 | <0.001 | 0.20 | 0.15–0.24 | 0.02 | <0.001 | 0.10 | 0.06–0.14 | 0.02 | <0.001 |
|  | Mean craniocaudal force (BW) | <0.001 | -0.10 | -0.12–(-0.09) | 0.01 | <0.001 | -0.06 | -0.07–(-0.04) | 0.01 | <0.001 | -0.05 | -0.07–(-0.03) | 0.01 | <0.001 |
|  | Peak vertical force (BW) | <0.001 | 0.30 | 0.24–0.35 | 0.03 | <0.001 | 0.17 | 0.11–0.22 | 0.03 | <0.001 | 0.13 | 0.07–0.18 | 0.03 | <0.001 |
|  | Vertical impulse (BWs) | <0.001 | 0.024 | 0.020–0.029 | 0.002 | <0.001 | 0.019 | 0.014–0.023 | 0.002 | <0.001 | 0.006 | 0.002–0.010 | 0.002 | 0.008 |
|  | Decelerative impulse (BWs)^a^ | <0.001 | -0.006 | -0.006–(-0.005) | 0.000 | <0.001 | -0.003 | -0.004–(-0.003) | 0.000 | <0.001 | -0.002 | -0.003–(-0.001) | 0.000 | <0.001 |
|  | Accelerative impulse (BWs) | <0.001 | -0.005 | -0.006–(-0.004) | 0.001 | <0.001 | -0.002 | -0.003–(-0.001) | 0.001 | 0.001 | -0.003 | -0.004–(-0.002) | 0.001 | <0.001 |
|  | Net craniocaudal impulse (BWs) | <0.001 | -0.010 | -0.012–(-0.009) | 0.001 | <0.001 | -0.005 | -0.007–(-0.004) | 0.001 | <0.001 | -0.005 | -0.007–(-0.003) | 0.001 | <0.001 |
|  | Direction of resultant force vector (°) | <0.001 | -3.4 | -3.9–(-2.9) | 0.3 | <0.001 | -1.7 | -2.2–(-1.2) | 0.2 | <0.001 | -1.7 | -2.2–(-1.2) | 0.2 | <0.001 |
| **All four limbs** | |  |  |  |  |  |  |  |  |  |  |  |  |  |
|  | Vertical impulse (BWs) | <0.001 | 0.052 | 0.043–0.060 | 0.004 | <0.001 | 0.038 | 0.030–0.046 | 0.004 | <0.001 | 0.014 | 0.006–0.022 | 0.004 | 0.001 |
|  | Net craniocaudal impulse (BWs) | <0.001 | -0.026 | -0.029–(-0.022) | 0.002 | <0.001 | -0.016 | -0.020–(-0.012) | 0.002 | <0.001 | -0.009 | -0.013–(-0.006) | 0.002 | <0.001 |
|  | Weight distribution (% of vertical impulse on FLs) | 0.340 | 0.2 | -0.5–0.9 | 0.3 | 0.532 | -0.3 | -0.9 –-0.4 | 0.3 | 0.419 | 0.5 | -0.2–1.1 | 0.3 | 0.145 |

CI = confidence interval, SE = standard error, BW = body weight
^a^ Lower values indicate greater deceleration.
