# Supplementary material for: Effect of bar jump height on kinetics and kinematics of take-off in agility dogs
Source: PLoS One. 2025 Jan 24;20(1):e0315907. doi: 10.1371/journal.pone.0315907 (PMC11761639; doi:10.1371/journal.pone.0315907)
Supplement: S2 Table — (DOCX) [file pone.0315907.s004.docx]

**S2 Table. Linear mixed model results: main effect of approach stride number on kinetics at take-off to a jump in agility dogs.**

|  | | **Stride number** | | | | **Estimated marginal mean ± SE** | |
| --- | --- | --- | --- | --- | --- | --- | --- |
| **Variable** | | **Estimate** | **95% CI** | **SE** | **p-value** | **One-stride approach** | **Two-stride approach** |
| **Forelimbs** | |  |  |  |  |  |  |
|  | Mean vertical force (BW) | 0.16 | 0.11–0.22 | 0.03 | <0.001 | 1.88 ± 0.03 | 1.72 ± 0.04 |
|  | Mean craniocaudal force (BW) | 0.07 | 0.04–0.10 | 0.02 | <0.001 | -0.01 ± 0.02 | -0.08 ± 0.02 |
|  | Peak vertical force (BW) | 0.00 | -0.14–0.14 | 0.07 | 0.980 | 3.05 ± 0.09 | 3.05 ± 0.10 |
|  | Vertical impulse (BWs) | 0.044 | 0.035–0.053 | 0.005 | <0.001 | 0.241 ± 0.005 | 0.197 ± 0.006 |
|  | Decelerative impulse (BWs)^a^ | 0.002 | -0.001–0.006 | 0.002 | 0.170 | -0.015 ± 0.002 | -0.017 ± 0.002 |
|  | Accelerative impulse (BWs) | 0.006 | 0.005–0.008 | 0.001 | <0.001 | 0.014 ± 0.001 | 0.007 ± 0.001 |
|  | Net craniocaudal impulse (BWs) | 0.009 | 0.004–0.013 | 0.002 | <0.001 | -0.001 ± 0.002 | -0.010 ± 0.003 |
|  | Direction of resultant force vector (°) | 2.5 | 1.5–3.5 | 0.5 | <0.001 | 89.9 ± 0.5 | 87.4 ± 0.6 |
| **Hindlimbs** | |  |  |  |  |  |  |
|  | Mean vertical force (BW) | 0.11 | 0.04–0.18 | 0.04 | 0.002 | 2.15 ± 0.05 | 2.04 ± 0.05 |
|  | Mean craniocaudal force (BW) | 0.06 | 0.04–0.09 | 0.01 | <0.001 | 0.17 ± 0.02 | 0.10 ± 0.02 |
|  | Peak vertical force (BW) | 0.27 | 0.18–0.37 | 0.05 | <0.001 | 3.81 ± 0.07 | 3.53 ± 0.07 |
|  | Vertical impulse (BWs) | 0.027 | 0.020–0.033 | 0.003 | <0.001 | 0.206 ± 0.003 | 0.179 ± 0.003 |
|  | Decelerative impulse (BWs)^a^ | 0.000 | -0.001–0.002 | 0.001 | 0.718 | -0.011 ± 0.001 | -0.011 ± 0.001 |
|  | Accelerative impulse (BWs) | 0.007 | 0.005–0.009 | 0.001 | <0.001 | 0.027 ± 0.001 | 0.020 ± 0.001 |
|  | Net craniocaudal impulse (BWs) | 0.007 | 0.005–0.010 | 0.001 | <0.001 | 0.016 ± 0.002 | 0.009 ± 0.002 |
|  | Direction of resultant force vector (°) | 1.5 | 0.7–2.3 | 0.4 | <0.001 | 94.4 ± 0.5 | 92.9 ± 0.5 |
| **All four limbs** | |  |  |  |  |  |  |
|  | Vertical impulse (BWs) | 0.068 | 0.055–0.081 | 0.007 | <0.001 | 0.447 ± 0.007 | 0.379 ± 0.008 |
|  | Net craniocaudal impulse (BWs) | 0.016 | 0.010–0.022 | 0.003 | <0.001 | 0.014 ± 0.003 | -0.002 ± 0.003 |
|  | Weight distribution (% of vertical impulse on FLs) | 1.4 | 0.3–2.5 | 0.5 | 0.011 | 53.9 ± 0.5 | 52.5 ± 0.6 |

CI = confidence interval, SE = standard error, BW = body weight
Estimate is reported as one-stride-approach - two-stride-approach.
