# Supplementary material for: Effect of bar jump height on kinetics and kinematics of take-off in agility dogs
Source: PLoS One. 2025 Jan 24;20(1):e0315907. doi: 10.1371/journal.pone.0315907 (PMC11761639; doi:10.1371/journal.pone.0315907)
Supplement: S3 Table — (DOCX) [file pone.0315907.s005.docx]

**S3 Table. Linear mixed model results: main effect of bar height and pairwise differences on jump arch and limb coordination at take-off to a jump in agility dogs.**

|  | | **Bar height** | **120% - 80%** | | | | **120%-100%** | | | | **100%-80%** | | | |
| --- | --- | --- | --- | --- | --- | --- | --- | --- | --- | --- | --- | --- | --- | --- |
| **Variable** | | **p-value** | **Estimate** | **95% CI** | **SE** | **p-value** | **Estimate** | **95% CI** | **SE** | **p-value** | **Estimate** | **95% CI** | **SE** | **p-value** |
| **Horizontal velocity at approach** (m/s) | | <0.001 | -0.41 | -0.49–(-0.33) | 0.04 | <0.001 | -0.29 | -0.37–(-0.21) | 0.04 | <0.001 | -0.12 | -0.20–(-0.04) | 0.04 | 0.003 |
| **Horizontal velocity after lift-off** (m/s) | | <0.001 | -0.69 | -0.79–(-0.59) | 0.05 | <0.001 | -0.51 | -0.61–(-0.41) | 0.05 | <0.001 | -0.18 | -0.28–(-0.08) | 0.05 | <0.001 |
| **Take-off distance** (cm) | | 0.012 | -9 | -15–(-2) | 3 | 0.009 | 0 | -7–6 | 3 | 0.920 | -8 | -15–(-2) | 3 | 0.010 |
| **Trunk angle at lift-off** (°) | | <0.001 | 5.8 | 5.0–6.6 | 0.4 | <0.001 | 4.0 | 3.3–4.8 | 0.4 | <0.001 | 1.8 | 1.0–2.6 | 0.4 | <0.001 |
| **Take-off angle** (°) | | <0.001 | 6.5 | 5.9–7.2 | 0.3 | <0.001 | 4.3 | 3.7–4.9 | 0.3 | <0.001 | 2.2 | 1.6–2.9 | 0.3 | <0.001 |
| **Trunk height at TrFL touch-down** (% of wither height) | | <0.001 | -2.6 | -3.3–(-2.0) | 0.3 | <0.001 | -1.5 | -2.1–(-0.9) | 0.3 | <0.001 | -1.1 | -1.7–(-0.5) | 0.3 | <0.001 |
| **Trunk height at the apex** (% of wither height) | | <0.001 | 39.4 | 36.4–42.4 | 1.5 | <0.001 | 26.6 | 23.7–29.5 | 1.5 | <0.001 | 12.8 | 9.8–15.8 | 1.5 | <0.001 |
| **Bar clearance** (% of wither height) | | <0.001 | -0.6 | -3.6–2.4 | 1.5 | 0.696 | 6.6 | 3.7–9.5 | 1.5 | <0.001 | -7.2 | -10.2–(-4.2) | 1.5 | <0.001 |
| **Stance time** | |  |  |  |  |  |  |  |  |  |  |  |  |  |
|  | Trailing forelimb (ms) | <0.001 | 9 | 7–11 | 1 | <0.001 | 7 | 5–9 | 1 | <0.001 | 2 | 0–4 | 1 | 0.058 |
|  | Leading forelimb (ms) | <0.001 | 5 | 3–7 | 1 | <0.001 | 3 | 1–6 | 1 | 0.002 | 2 | 1–4 | 1 | 0.147 |
|  | Trailing hindlimb (ms) | <0.001 | 5 | 4–7 | 1 | <0.001 | 4 | 3–6 | 1 | <0.001 | 1 | -1–2 | 1 | 0.196 |
|  | Leading hindlimb (ms) | <0.001 | 6 | 3–8 | 1 | <0.001 | 5 | 3–7 | 1 | <0.001 | 1 | -1–2 | 1 | 0.538 |
| **Synchronicity** | |  |  |  |  |  |  |  |  |  |  |  |  |  |
|  | Forelimbs (% of TrFL stance time)^a^ | <0.001 | -6.1 | -8.3–(-3.8) | 1.1 | <0.001 | -3.8 | -6.0–(-1.6) | 1.1 | <0.001 | -2.3 | -4.5–0.0 | 1.1 | 0.048 |
|  | Hindlimbs (% of TrHL stance time)^a^ | <0.001 | -9.6 | -11.8–(-7.5) | 1.1 | <0.001 | -6.6 | -8.7–(-4.5) | 1.1 | <0.001 | -3.0 | -5.1–(-0.9) | 1.1 | 0.006 |
| **Distance between limbs at TD** | |  |  |  |  |  |  |  |  |  |  |  |  |  |
|  | Craniocaudal distance between FLs (cm) | <0.001 | -3.5 | -4.8–(-2.2) | 0.7 | <0.001 | -2.1 | 3.4–(-0.9) | 0.6 | 0.001 | -1.4 | -2.6–(-0.1) | 0.6 | 0.037 |
|  | Craniocaudal distance between HLs (cm) | <0.001 | -6.2 | -7.5–(-4.9) | 0.7 | <0.001 | -4.2 | -5.5–(-3.0) | 0.6 | <0.001 | -2.0 | -3.3–(-0.7) | 0.6 | 0.003 |
|  | Craniocaudal distance between LeFL and TrHL (cm) | <0.001 | -5.1 | -6.6–(-3.7) | 0.7 | <0.001 | -3.6 | -5.0–(-2.2) | 0.7 | <0.001 | -1.5 | -2.9–(-0.1) | 0.7 | 0.039 |
|  | Mediolateral distance between FLs (cm) | <0.001 | 1.4 | 0.8–2.0 | 0.3 | <0.001 | 1.0 | 0.4-1.6 | 0.3 | <0.001 | 0.4 | -0.2–1.0 | 0.3 | 0.172 |
|  | Mediolateral distance between HLs (cm) | <0.001 | 1.5 | 1.1–1.9 | 0.2 | <0.001 | 1.1 | 0.7–1.5 | 0.2 | <0.001 | 0.4 | 0.0–0.8 | 0.2 | 0.063 |
|  | Craniocaudal distance between TrHL and trunk marker (cm) | <0.001 | -1.9 | -2.5–(-1.2) | 0.3 | <0.001 | -1.5 | -2.1–(-0.8) | 0.3 | <0.001 | -0.4 | -1.0–0.3 | 0.3 | 0.230 |
|  | Craniocaudal distance between LeHL and trunk marker (cm) | <0.001 | -1.8 | -2.6–(-1.0) | 0.4 | <0.001 | -1.3 | -2.1–(-0.5) | 0.4 | 0.001 | -0.5 | -1.3–0.3 | 0.4 | 0.210 |
| **Limb angle** | |  |  |  |  |  |  |  |  |  |  |  |  |  |
|  | TrFL at touch-down (°) | <0.001 | -4.3 | -5.2–(-3.3) | 0.5 | <0.001 | -2.8 | -3.7–(-1.9) | 0.5 | <0.001 | -1.5 | -2.4–(-0.6) | 0.5 | 0.002 |
|  | TrFL at lift-off (°) | <0.001 | -3.1 | -3.8–(-2.3) | 0.4 | <0.001 | -1.9 | -2.6–(-1.1) | 0.4 | <0.001 | -1.2 | -1.9–(-0.4) | 0.4 | 0.003 |
|  | LeFL at touch-down (°) | <0.001 | -3.9 | -4.7–(-3.0) | 0.5 | <0.001 | -2.4 | -3.3–(-1.5) | 0.4 | <0.001 | -1.5 | -2.3–(-0.6) | 0.4 | 0.001 |
|  | LeFL at lift-off (°) | <0.001 | -6.2 | -7.2–(-5.1) | 0.5 | <0.001 | -4.0 | -5.0–(-3.1) | 0.5 | <0.001 | -2.1 | -3.1–(-1.1) | 0.5 | <0.001 |
|  | TrHL at touch-down (°) | <0.001 | -3.6 | -4.5–(-2.8) | 0.4 | <0.001 | -2.8 | -3.6–(-2.0) | 0.4 | <0.001 | -0.8 | -1.6–0.0 | 0.4 | 0.053 |
|  | TrHL at lift-off (°) | <0.001 | -5.5 | -6.3–(-4.7) | 0.4 | <0.001 | -3.4 | -4.2–(-2.6) | 0.4 | <0.001 | -2.1 | -2.9–(-1.3) | 0.4 | <0.001 |
|  | LeHL at touch-down (°) | <0.001 | -2.3 | -3.2–(-1.3) | 0.5 | <0.001 | -1.7 | -2.7–(-0.8) | 0.5 | <0.001 | -0.6 | -1.5–0.4 | 0.5 | 0.255 |
|  | LeHL at lift-off (°) | <0.001 | -4.9 | -5.7–(-4.0) | 0.4 | <0.001 | -2.5 | -3.3–(-1.7) | 0.4 | <0.001 | -2.4 | -3.2–(-1.6) | 0.4 | <0.001 |

CI = confidence interval, SE = standard error, TD = touch-down, TrFL = trailing forelimb, LeFL = leading forelimb, TrHL = trailing hindlimb, LeHL = leading hindlimb
^a^ Lower values indicate greater synchronicity.
