# Supplementary material for: Effect of bar jump height on kinetics and kinematics of take-off in agility dogs
Source: PLoS One. 2025 Jan 24;20(1):e0315907. doi: 10.1371/journal.pone.0315907 (PMC11761639; doi:10.1371/journal.pone.0315907)
Supplement: S4 Table — (DOCX) [file pone.0315907.s006.docx]

**S4 Table. Linear mixed model results: main effect of approach stride number on jump arch and limb coordination at take-off to a jump in agility dogs.**

|  | | **Stride number** | | | | **Estimated marginal mean ± SE** | |
| --- | --- | --- | --- | --- | --- | --- | --- |
| **Variable** | | **Estimate** | **95% CI** | **SE** | **p-value** | **One-stride approach** | **Two-stride approach** |
| **Horizontal velocity at approach** (m/s) | | 0.55 | 0.38–0.72 | 0.08 | <0.001 | 7.37 ± 0.11 | 6.82 ± 0.12 |
| **Horizontal velocity after lift-off** (m/s) | | 0.36 | 0.18–0.53 | 0.09 | <0.001 | 7.18 ± 0.14 | 6.82 ± 0.15 |
| **Take-off distance** (cm) | | 74 | 62–85 | 6 | <0.001 | 223 ± 9 | 149 ± 9 |
| **Trunk angle at lift-off** (°) | | 2.3 | 0.9–3.6 | 0.7 | 0.001 | 18.7 ± 0.7 | 16.4 ± 0.8 |
| **Take-off angle** (°) | | 1.0 | -0.1–2.2 | 0.6 | 0.071 | 14.1 ± 0.5 | 13.0 ± 0.6 |
| **Trunk height at TrFL touch-down** (% of wither height) | | -1.5 | -2.6–(-0.3) | 0.6 | 0.017 | 75.2 ± 0.7 | 75.7 ± 0.7 |
| **Trunk height at the apex** (% of wither height) | | 17.3 | 12.5–22.2 | 2.5 | <0.001 | 155.0 ± 3.4 | 137.7 ± 3.4 |
| **Bar clearance** (% of wither height) | | 17.3 | 12.5–22.2 | 2.5 | <0.001 | 55.0 ± 3.2 | 37.7 ± 3.5 |
| **Stance time** | |  |  |  |  |  |  |
|  | Trailing forelimb (ms) | 0 | -3–4 | 2 | 0.799 | 85 ± 3 | 85 ± 3 |
|  | Leading forelimb (ms) | 9 | 5–12 | 2 | <0.001 | 82 ± 2 | 73 ± 3 |
|  | Trailing hindlimb (ms) | 1 | -2–3 | 1 | 0.524 | 81 ± 2 | 81 ± 2 |
|  | Leading hindlimb (ms) | 0 | -3–3 | 2 | 0.778 | 76 ± 2 | 77 ± 2 |
| **Synchronicity** | |  |  |  |  |  |  |
|  | Forelimbs (% of TrFL stance time)^a^ | 4.7 | 1.1–8.3 | 1.8 | 0.010 | 56.0 ± 1.9 | 51.3 ± 2.1 |
|  | Hindlimbs (% of TrHL stance time)^a^ | 6.9 | 3.3–10.4 | 1.8 | <0.001 | 22.0 ± 2.4 | 15.1 ± 2.6 |
| **Distance between limbs at TD** | |  |  |  |  |  |  |
|  | Craniocaudal distance between FLs (cm) | 7.2 | 4.8–9.5 | 1.2 | <0.001 | 37.2 ± 1.3 | 30.0 ± 1.4 |
|  | Craniocaudal distance between HLs (cm) | 5.6 | 3.2–8.0 | 1.2 | <0.001 | 13.5 ± 1.5 | 8.0 ± 1.6 |
|  | Craniocaudal distance between LeFL and TrHL (cm) | 9.3 | 6.7–11.9 | 1.3 | <0.001 | 15.0 ± 1.2 | 5.7 ± 1.4 |
|  | Mediolateral distance between FLs (cm) | -0.4 | -1.5–0.7 | 0.6 | 0.462 | 8.4 ± 0.7 | 8.8 ± 0.7 |
|  | Mediolateral distance between HLs (cm) | -1.7 | -2.5–(-1.0) | 0.4 | <0.001 | 12.4 ± 0.5 | 14.2 ± 0.5 |
|  | Craniocaudal distance between TrHL and trunk marker (cm) | -2.1 | -3.3–(-0.9) | 0.6 | 0.001 | 4.5 ± 0.6 | 6.5 ± 0.6 |
|  | Craniocaudal distance between LeHL and trunk marker (cm) | -3.5 | -4.8–(-2.2) | 0.6 | <0.001 | 3.4 ± 0.5 | 6.9 ± 0.6 |
| **Limb angle** | |  |  |  |  |  |  |
|  | TrFL at touch-down (°) | -2.5 | -4.1–(-0.8) | 0.9 | 0.005 | 69.7 ± 0.9 | 72.1 ± 1.0 |
|  | TrFL at lift-off (°) | 5.9 | 4.6–7.3 | 0.7 | <0.001 | 132.6 ± 0.8 | 126.6 ± 0.8 |
|  | LeFL at touch-down (°) | -3.1 | -4.5–(-1.7) | 0.7 | <0.001 | 68.1 ± 0.6 | 71.2 ± 0.7 |
|  | LeFL at lift-off (°) | 6.2 | 4.5–7.9 | 0.9 | <0.001 | 119.0 ± 1.0 | 112.8 ± 1.1 |
|  | TrHL at touch-down (°) | -1.6 | -3.0–(-0.2) | 0.7 | 0.030 | 64.3 ± 0.6 | 65.9 ± 0.7 |
|  | TrHL at lift-off (°) | 3.7 | 2.3–5.0 | 0.7 | <0.001 | 126.6 ± 0.7 | 122.9 ± 0.8 |
|  | LeHL at touch-down (°) | -3.4 | -5.0–(-1.8) | 0.8 | <0.001 | 62.9 ± 0.7 | 66.4 ± 0.8 |
|  | LeHL at lift-off (°) | 1.7 | 0.3–3.0 | 0.7 | 0.018 | 122.3 ± 0.9 | 120.7 ± 0.9 |

CI = confidence interval, SE = standard error, TD = touch-down, TrFL = trailing forelimb, LeFL = leading forelimb, TrHL = trailing hindlimb, LeHL = leading hindlimb
Estimate is reported as one-stride-approach - two-stride-approach.

^a^ Lower values indicate greater synchronicity.
