# Supplementary material for: Effect of bar jump height on kinetics and kinematics of take-off in agility dogs
Source: PLoS One. 2025 Jan 24;20(1):e0315907. doi: 10.1371/journal.pone.0315907 (PMC11761639; doi:10.1371/journal.pone.0315907)
Supplement: S5 Table — (DOCX) [file pone.0315907.s007.docx]

**S5 Table. Linear mixed model results: main effect of bar height and pairwise differences in sagittal joint kinematics at take-off to a jump in agility dogs.**

|  | | **Bar height** | **120% - 80%** | | | | **120%-100%** | | | | **100%-80%** | | | |
| --- | --- | --- | --- | --- | --- | --- | --- | --- | --- | --- | --- | --- | --- | --- |
| **Variable** | | **p-value** | **Estimate** | **95% CI** | **SE** | **p-value** | **Estimate** | **95% CI** | **SE** | **p-value** | **Estimate** | **95% CI** | **SE** | **p-value** |
| **Trailing forelimb** | |  |  |  |  |  |  |  |  |  |  |  |  |  |
|  | Shoulder peak flexion (°) | 0.23 | -0.9 | -1.9–0.1 | 0.5 | 0.092 | -0.5 | -1.6–0.5 | 0.5 | 0.287 | -0.3 | -1.4–0.7 | 0.5 | 0.511 |
|  | Shoulder peak extension (°) | <0.001 | 3.0 | 1.8–4.1 | 0.6 | <0.001 | 2.0 | 0.8–3.1 | 0.6 | 0.001 | 1.0 | -0.2–2.2 | 0.6 | 0.091 |
|  | Shoulder ROM (°) | <0.001 | 3.8 | 2.5–5.2 | 0.7 | <0.001 | 2.5 | 1.1–3.8 | 0.7 | <0.001 | 1.4 | 0.0–2.7 | 0.7 | 0.054 |
|  | Elbow peak flexion (°) | <0.001 | -5.3 | -6.9–(-3.7) | 0.8 | <0.001 | -3.6 | -5.2–(-2.0) | 0.8 | <0.001 | -1.7 | -3.4–(-0.1) | 0.8 | 0.034 |
|  | Elbow peak extension (°) | 0.014 | -1.9 | -3.1–(-0.6) | 0.6 | 0.004 | -0.8 | -2.0–0.4 | 0.6 | 0.191 | -1.1 | -2.3–0.2 | 0.6 | 0.093 |
|  | Elbow ROM (°) | <0.001 | 3.4 | 2.0–4.7 | 0.7 | <0.001 | 2.7 | 1.4–4.0 | 0.7 | <0.001 | 0.7 | -0.6–2.0 | 0.7 | 0.297 |
|  | Carpus peak flexion (°) | 0.872 | 0.2 | -1.7–2.0 | 0.9 | 0.859 | 0.5 | -1.3–2.2 | 0.9 | 0.607 | -0.3 | -2.1–1.5 | 0.9 | 0.747 |
|  | Carpus peak extension (°) | 0.002 | 3.4 | 1.5–5.3 | 1.0 | <0.001 | 2.5 | 1.5–5.3 | 1.0 | 0.008 | 0.9 | -1.0–2.8 | 1.0 | 0.365 |
|  | Carpus ROM (°) | 0.007 | 3.3 | 1.2–5.4 | 1.0 | 0.002 | 2.1 | 0.1–4.1 | 1.0 | 0.039 | 1.2 | -0.8–3.3 | 1.0 | 0.244 |
| **Leading forelimb** | |  |  |  |  |  |  |  |  |  |  |  |  |  |
|  | Shoulder peak flexion (°) | 0.040 | 0.9 | 0.3–2.3 | 0.5 | 0.085 | -0.4 | -1.4–0.6 | 0.5 | 0.428 | 1.3 | 0.3–2.3 | 0.5 | 0.0125 |
|  | Shoulder peak extension (°) | 0.355 | 0.9 | -0.4–2.1 | 0.6 | 0.159 | 0.6 | -0.6–1.8 | 0.6 | 0.342 | 0.3 | -0.9–1.5 | 0.6 | 0.617 |
|  | Shoulder ROM (°) | 0.151 | 0.1 | -1.0–1.2 | 0.6 | 0.864 | 1.0 | -0.1–2.0 | 0.5 | 0.077 | -0.9 | -1.9–0.2 | 0.5 | 0.120 |
|  | Elbow peak flexion (°) | 0.180 | -0.3 | -2.1–1.4 | 0.9 | 0.708 | -1.5 | -3.2–0.2 | 0.8 | 0.078 | 1.2 | -0.5–2.9 | 0.9 | 0.180 |
|  | Elbow peak extension (°) | 0.343 | 0.3 | -0.9–1.6 | 0.6 | 0.591 | -0.6 | -1.8–0.6 | 0.6 | 0.354 | 0.9 | -0.3–2.1 | 0.6 | 0.151 |
|  | Elbow ROM (°) | 0.460 | 0.7 | -0.8–2.2 | 0.8 | 0.388 | 0.9 | -0.6–2.3 | 0.7 | 0.229 | -0.2 | -1.7–1.3 | 0.8 | 0.761 |
|  | Carpus peak flexion (°) | 0.413 | -0.8 | -2.5–0.8 | 0.8 | 0.328 | -1.0 | -2.6–0.6 | 0.8 | 0.205 | 0.2 | -1.4–1.8 | 0.8 | 0.800 |
|  | Carpus peak extension (°) | 0.017 | 2.3 | 0.3–4.4 | 1.0 | 0.027 | -0.5 | -2.5–1.5 | 1.0 | 0.628 | 2.8 | 0.8–4.9 | 1.0 | 0.007 |
|  | Carpus ROM (°) | 0.018 | 3.1 | 0.8–5.4 | 1.2 | 0.008 | 0.5 | -1.7–2.7 | 1.1 | 0.637 | 2.6 | 0.3–4.8 | 1.1 | 0.025 |

| **Trailing hindlimb** | |  |  |  |  |  |  |  |  |  |  |  |  |  |
| --- | --- | --- | --- | --- | --- | --- | --- | --- | --- | --- | --- | --- | --- | --- |
|  | Hip peak flexion (°) | <0.001 | -2.4 | -3.9–(-1.0) | 0.7 | <0.001 | -2.3 | -3.7–(-0.9) | 0.7 | 0.001 | -0.1 | -1.5–1.3 | 0.7 | 0.859 |
|  | Hip peak extension (°) | 0.625 | 0.7 | -0.7–2.1 | 0.7 | 0.333 | 0.3 | -1.1–1.7 | 0.7 | 0.649 | 0.4 | -1.0–1.8 | 0.7 | 0.589 |
|  | Hip ROM (°) | <0.001 | 3.2 | 1.9–4.4 | 0.6 | <0.001 | 2.6 | 1.4–3.8 | 0.6 | <0.001 | 0.5 | -0.7–1.8 | 0.6 | 0.377 |
|  | Stifle peak flexion (°) | 0.647 | -0.6 | -2.4–1.3 | 0.9 | 0.541 | -0.8 | -2.6–1.0 | 0.9 | 0.360 | 0.3 | -1.6–2.1 | 0.9 | 0.779 |
|  | Stifle peak extension (°) | <0.001 | 1.7 | 0.8–2.6 | 0.4 | <0.001 | 1.6 | 0.7–2.4 | 0.4 | <0.001 | 0.1 | -0.7–1.0 | 0.4 | 0.754 |
|  | Stifle ROM (°) | 0.001 | 2.4 | 0.9–3.9 | 0.8 | 0.002 | 2.4 | 1.0–3.9 | 0.7 | 0.001 | 0.0 | -1.5–1.4 | 0.7 | 0.958 |
|  | Tarsus peak flexion (°) | <0.001 | -4.6 | -6.5–(-2.8) | 0.9 | <0.001 | -3.1 | -4.9–(-1.3) | 0.9 | <0.001 | -1.6 | -3.4–0.3 | 0.9 | 0.092 |
|  | Tarsus peak extension (°) | 0.945 | -0.1 | -1.0–0.8 | 0.5 | 0.792 | 0.0 | -0.9–0.9 | 0.4 | 0.964 | -0.1 | -1.0–0.8 | 0.5 | 0.753 |
|  | Tarsus ROM (°) | <0.001 | 4.5 | 2.8–6.2 | 0.8 | <0.001 | 3.1 | 1.5–4.8 | 0.8 | <0.001 | 1.4 | -0.3–3.0 | 0.8 | 0.102 |
| **Leading hindlimb** | |  |  |  |  |  |  |  |  |  |  |  |  |  |
|  | Hip peak flexion (°) | 0.172 | 1.4 | -0.1–3.0 | 0.8 | 0.067 | 0.4 | -1.1–1.9 | 0.8 | 0.582 | 1.0 | -0.5–2.6 | 0.8 | 0.190 |
|  | Hip peak extension (°) | 0.022 | 2.2 | 0.6–3.7 | 0.8 | 0.006 | 1.2 | -0.3–2.7 | 0.8 | 0.108 | 0.9 | -0.6–2.5 | 0.8 | 0.227 |
|  | Hip ROM (°) | 0.534 | 0.7 | -0.9-2.3 | 0.8 | 0.397 | 0.8 | -0.7–2.4 | 0.8 | 0.292 | -0.1 | -1.7–1.4 | 0.8 | 0.857 |
|  | Stifle peak flexion (°) | <0.001 | -2.0 | -3.4–(-0.5) | 0.7 | 0.009 | -2.8 | -4.2–(-1.4) | 0.7 | <0.001 | 0.8 | -0.7–2.3 | 0.7 | 0.278 |
|  | Stifle peak extension (°) | 0.857 | -0.2 | -1.1–0.7 | 0.4 | 0.630 | 0.0 | -0.8–0.8 | 0.4 | 1.000 | -0.2 | -1.1–0.7 | 0.4 | 0.627 |
|  | Stifle ROM (°) | <0.001 | 1.8 | 0.6–2.9 | 0.6 | 0.002 | 2.8 | 1.7–3.9 | 0.6 | <0.001 | -1.0 | -2.1–0.1 | 0.6 | 0.083 |
|  | Tarsus peak flexion (°) | <0.001 | -2.4 | -4.4–(-0.5) | 1.0 | 0.016 | -3.9 | -5.8–(-2.0) | 1.0 | <0.001 | 1.5 | -0.5–3.4 | 1.0 | 0.133 |
|  | Tarsus peak extension (°) | 0.002 | 1.7 | 0.8–3.6 | 0.5 | <0.001 | 0.9 | 0.0–1.8 | 0.5 | 0.045 | 0.8 | -0.2–1.7 | 0.5 | 0.107 |
|  | Tarsus ROM (°) | <0.001 | 4.1 | 2.3–6.0 | 0.9 | <0.001 | 4.8 | 3.0–6.6 | 0.9 | <0.001 | -0.7 | -2.5–1.2 | 0.9 | 0.463 |

CI = confidence interval, SE = standard error, ROM = range of motion
