# Supplementary material for: Effect of bar jump height on kinetics and kinematics of take-off in agility dogs
Source: PLoS One. 2025 Jan 24;20(1):e0315907. doi: 10.1371/journal.pone.0315907 (PMC11761639; doi:10.1371/journal.pone.0315907)
Supplement: S6 Table — (DOCX) [file pone.0315907.s008.docx]

**S6 Table. Linear mixed model results: main effect of approach stride number on sagittal joint kinematics at take-off to a jump in agility dogs.**

|  | | **Stride number** | | | | **Estimated marginal mean ± SE** | |
| --- | --- | --- | --- | --- | --- | --- | --- |
| **Variable** | | **Estimate** | **95% CI** | **SE** | **p-value** | **One-stride approach** | **Two-stride approach** |
| **Trailing forelimb** | |  |  |  |  |  |  |
|  | Shoulder peak flexion (°) | -5.9 | -7.8–(-3.9) | 1.0 | <0.001 | 111.6 ± 1.5 | 117.5 ± 1.6 |
|  | Shoulder peak extension (°) | -6.0 | -8.30–(-3.8) | 1.1 | <0.001 | 126.5 ± 2.1 | 132.6 ± 2.2 |
|  | Shoulder ROM (°) | -0.5 | -3.1–2.0 | 1.3 | 0.672 | 14.8 ± 1.4 | 15.3 ± 1.6 |
|  | Elbow peak flexion (°) | -4.6 | -7.6–(-1.6) | 1.5 | 0.003 | 119.8 ± 2.0 | 124.4 ± 2.2 |
|  | Elbow peak extension (°) | -1.7 | -4.0–0.5 | 1.1 | 0.134 | 157.6 ± 1.2 | 159.3 ± 1.3 |
|  | Elbow ROM (°) | 2.2 | -0.1–4.4 | 1.1 | 0.058 | 37.2 ± 1.0 | 35.0 ± 1.1 |
|  | Carpus peak flexion (°) | 0.6 | -2.9–4.0 | 1.7 | 0.746 | 184.7 ± 2.3 | 184.2 ± 2.4 |
|  | Carpus peak extension (°) | -2.3 | -6.0–1.3 | 1.9 | 0.213 | 235.0 ± 2.9 | 237.3 ± 3.1 |
|  | Carpus ROM (°) | -2.6 | -6.1–0.9 | 1.8 | 0.148 | 50.6 ± 1.5 | 53.2 ± 1.8 |
| **Leading forelimb** | |  |  |  |  |  |  |
|  | Shoulder peak flexion (°) | -1.2 | -3.3–0.9 | 1.1 | 0.251 | 118.9 ± 1.9 | 120.1 ± 2.0 |
|  | Shoulder peak extension (°) | 1.7 | -0.8–4.3 | 1.3 | 0.183 | 141.9 ± 2.1 | 140.1 ± 2.2 |
|  | Shoulder ROM (°) | 4.0 | 1.9–6.0 | 1.0 | <0.001 | 23.3 ± 0.9 | 19.4 ± 1.1 |
|  | Elbow peak flexion (°) | -5.5 | -9.0–(-2.1) | 1.8 | 0.002 | 122.1 ± 2.4 | 127.7 ± 2.5 |
|  | Elbow peak extension (°) | 0.7 | -1.7–3.1 | 1.2 | 0.557 | 163.3 ± 1.3 | 162.6 ± 1.4 |
|  | Elbow ROM (°) | 6.2 | 3.3–9.1 | 1.5 | <0.001 | 41.0 ± 1.6 | 34.8 ± 1.7 |
|  | Carpus peak flexion (°) | 1.9 | -1.1–5.0 | 1.5 | 0.218 | 188.1 ± 1.9 | 186.2 ± 2.1 |
|  | Carpus peak extension (°) | 11.6 | 7.7–15.5 | 2.0 | <0.001 | 237.7 ± 3.1 | 226.1 ± 3.2 |
|  | Carpus ROM (°) | 9.0 | 4.9–13.1 | 2.1 | <0.001 | 49.5 ± 2.1 | 40.5 ± 2.4 |
| **Trailing hindlimb** | |  |  |  |  |  |  |
|  | Hip peak flexion (°) | 5.8 | 3.2–8.5 | 1.4 | <0.001 | 131.9 ± 1.9 | 126.1 ± 2.0 |
|  | Hip peak extension (°) | 4.0 | 1.4–6.6 | 1.3 | 0.003 | 173.9 ± 1.5 | 169.9 ± 1.7 |
|  | Hip ROM (°) | -1.7 | -3.9–0.6 | 1.1 | 0.141 | 42.0 ± 1.1 | 43.6 ± 1.3 |
|  | Stifle peak flexion (°) | -0.6 | -3.7–2.6 | 1.6 | 0.722 | 130.0 ± 1.4 | 130.6 ± 1.6 |
|  | Stifle peak extension (°) | -0.1 | -1.7–1.6 | 0.8 | 0.949 | 156.5 ± 1.3 | 156.6 ± 1.4 |
|  | Stifle ROM (°) | 1.9 | -0.4–4.2 | 1.1 | 0.107 | 27.0 ± 0.8 | 25.1 ± 1.0 |
|  | Tarsus peak flexion (°) | -6.5 | -9.9–(-3.2) | 1.7 | <0.001 | 102.2 ± 1.8 | 108.8 ± 2.0 |
|  | Tarsus peak extension (°) | 0.5 | -1.3–2.2 | 0.9 | 0.595 | 174.7 ± 1.3 | 174.2 ± 1.4 |
|  | Tarsus ROM (°) | 7.0 | 4.2–9.8 | 1.4 | <0.001 | 72.5 ± 1.2 | 65.5 ± 1.4 |
| **Leading hindlimb** | |  |  |  |  |  |  |
|  | Hip peak flexion (°) | 0.7 | -2.1–3.5 | 1.4 | 0.618 | 132.4 ± 2.4 | 131.7 ± 2.5 |
|  | Hip peak extension (°) | -2.8 | -5.6–0.1 | 1.4 | 0.054 | 167.9 ± 2.4 | 170.6 ± 2.5 |
|  | Hip ROM (°) | -3.6 | -6.3–(-0.9) | 1.4 | 0.010 | 35.4 ± 1.4 | 39.0 ± 1.6 |
|  | Stifle peak flexion (°) | 2.1 | -0.4–4.7 | 1.3 | 0.101 | 135.7 ± 1.4 | 133.6 ± 1.6 |
|  | Stifle peak extension (°) | 1.9 | 0.3–3.5 | 0.8 | 0.021 | 161.9 ± 1.3 | 160.1 ± 1.4 |
|  | Stifle ROM (°) | -0.2 | -2.1–1.6 | 0.9 | 0.801 | 26.2 ± 0.8 | 26.4 ± 1.0 |
|  | Tarsus peak flexion (°) | -1.3 | -4.7–2.0 | 1.7 | 0.437 | 106.8 ± 1.7 | 108.1 ± 1.9 |
|  | Tarsus peak extension (°) | 0.8 | -0.9–2.4 | 0.8 | 0.350 | 176.4 ± 1.0 | 175.6 ± 1.1 |
|  | Tarsus ROM (°) | 2.2 | -1.0–5.4 | 1.6 | 0.178 | 69.6 ± 1.6 | 67.6 ± 1.8 |

CI = confidence interval, SE = standard error, ROM = range of motion

Estimate is reported as one-stride approach - two-stride approach.
